# Green Synthesis of Gold Nanoparticles Capped with Procyanidins from *Leucosidea sericea* as Potential Antidiabetic and Antioxidant Agents

**DOI:** 10.3390/biom10030452

**Published:** 2020-03-13

**Authors:** Umar M. Badeggi, Enas Ismail, Adewale O. Adeloye, Subelia Botha, Jelili A. Badmus, Jeanine L. Marnewick, Christopher N. Cupido, Ahmed A. Hussein

**Affiliations:** 1 Chemistry Department, Cape Peninsula University of Technology, Symphony Rd., Bellville 7535, South Africa; 217064221@mycput.ac.za (U.M.B.); enas.ismail4@yahoo.com (E.I.); aowale@gmail.com (A.O.A.); 2 Electron Microscope Unit, University of the Western Cape, Bellville 7535, South Africa; subotha@uwc.ac.za; 3 Oxidative Stress Research Centre, Institute of Biomedical and Microbial Biotechnology, Cape Peninsula University of Technology, Symphony Rd., Bellville 7535, South Africa; jabadmus@lautech.edu.ng (J.A.B.); Marnewickj@cput.ac.za (J.L.M.); 4 Department of Botany, University of Fort Hare, Private Bag X1314, Alice 5700, South Africa; ccupido@ufh.ac.za

**Keywords:** *Leucosidea sericea*, alpha-amylase, alpha-glucosidase, diabetics, gold nanoparticles

## Abstract

In this study, procyanidins fractions of dimers and trimers (F1–F2) from the *Leucosidea sericea* total extract (LSTE) were investigated for their chemical constituents. The total extract and the procyanidins were employed in the synthesis of gold nanoparticles (Au NPs) and fully characterized. Au NPs of 6, 24 and 21 nm were obtained using LSTE, F1 and F2 respectively. Zeta potential and in vitro stability studies confirmed the stability of the particles. The enzymatic activity of LSTE, F1, F2 and their corresponding Au NPs showed strong inhibitory alpha-amylase activity where F1 Au NPs demonstrated the highest with IC_50_ of 1.88 µg/mL. On the other hand, F2 Au NPs displayed the strongest alpha-glucosidase activity at 4.5 µg/mL. F2 and F2 Au NPs also demonstrated the highest antioxidant activity, 1834.0 ± 4.7 μM AAE/g and 1521.9 ± 3.0 μM TE/g respectively. The study revealed not only the ability of procyanidins dimers (F1 and F2) in forming biostable and bioactive Au NPs but also, a significant enhancement of the natural products activities, which could improve the smart delivery in future biomedical applications.

## 1. Introduction

Unlike the physical and chemical protocols, the green synthesis offers a variety of advantages in line with the principles of green chemistry [1]. For instance, the resources employed are often plant materials that are readily available, accessible and affordable [2]. Plants contain complex structures that can be used in the reduction and stabilization of the nanoparticles, requiring no external stabilizers [3]. The solvents employed are usually eco-friendly, making the environment safe from remnants of toxic chemicals, and the products are comparatively less toxic [4]. Other advantages include cost-effectiveness as sophisticated instrumentation is not required [5]. Small quantities (in grams) from part of the plant, e.g., the leaves, are usually enough for synthesis [5,6]. Furthermore, the synthesis is generally fast [6]. It takes a few minutes to a couple of hours and the new materials have exciting characteristics that had made them the focus of modern-day research [7]. It was thought that their increased surface area, size and shape contributed to their properties [8,9]. Among other great benefits of synthesizing and applying nanoparticles through the green route is their biocompatibility [5]. Many green synthesized nanoparticles have demonstrated interesting biological activities [5,10] including antimicrobial and antidiabetic properties.

Diabetes mellitus has undoubtedly become a serious health challenge. More worrisome is type II diabetes mellitus (T2DM), which accounts for 90% of diabetes mellitus. It occurs as a result of the inefficient processing of insulin [11]. As of 2017, the population of adults between the ages of 20 and 79 that suffered from diabetes was 425 million [12]. This is equivalent to 9.9% of the world’s population [12]. By estimation, this disease would have drastically increased by 48% in 28 years if not properly managed [13]. Although drugs like miglitol, vigliobose as well as acarbose are available in the market, they are costly and their continuous use is associated with side effects like diarrhea, dropsy, heart failure, damage to the liver, weight gain, abdominal pain, hyperglycemia, and flatulence, necessitating the need for more potent and newer remedies [14,15,16].

It is well established that bioactive compounds in various plants possess significant effects in delaying and management of T2DM [17]. Extracts from different plants have been reported as alpha-glucosidase and alpha-amylase inhibitors [18,19,20,21,22,23,24,25,26,27]. Additionally, biologically synthesized Au NPs using plant extracts showed interesting antidiabetic activity. The extracts of *Chamalcostus cuspidatus* [28], *Gymnema sylvestre* [29], *Cassia fistula* stem bark [30], *Hericium erinaceus* [31], *Turbinaria conoides* [32], *Sambucus nigra* [33] and *Sargassum swartzi* [34] displayed antidiabetic activities in various investigations. Silver/gold NPs of *Ocimum basilicum* [35] and cinnamon extract [36] also lowers glucose levels. The use of single molecules as reducing/stabilizing agents for nanoparticle formation with certain bioactivity has been reported. Plant polyphenols are among the preferred candidates [37,38]. It has been shown that gallic and protocatechuic acids possess reducing abilities in forming nanoparticles [39,40,41,42]. Hesperidin [43,44], diosmin, and naringin [44], curcumin [45], guavanoic acid [46], phloridzin, an antidiabetic agent found in fruits and its aglycon [47], escin [48], resveratrol [49], and gymnemic acid [50] were found to be responsible for the biosynthesis of Au NPs. Other compounds that have been used in the formation of Au NPs with antidiabetic properties include chitosan, chondroitin sulfate, tyrosine and tryptophan [51,52,53].

Like the metal-reducing ability of plant extracts, the antioxidants activity has been associated with the plants’ phenolics [54]. Pu and colleagues [55] argued that antioxidants possess free radical scavenging properties, hence, they play role in promoting health and preventing diseases. In 2019 alone, several antioxidant activities [56,57,58,59,60,61,62,63] have been carried out on gold nanoparticles through the green route. The results demonstrated encouraging scavenging activities.

*Leucosidea sericea* (Rosaceae family) is the only species belonging to the genus *Leucosidea* in the Southern part of Africa [64]. Family Rosaceae consists of approximately 300 species out of which only nine are native to Southern African countries like Zimbabwe, Lesotho and South Africa. It is a very popular plant among the South Africans with names like ‘Ouhout’ by the Afrikaans and ‘umTshitshi’ according to the Zulu people [65]. Traditionally, it is used as protection against charm, vermifuge, astringent, for expelling parasitic worms and as a treatment for ophthalmia [66,67]. Extracts from this economic plant have been prepared from different solvents and have shown anti-inflammatory, antioxidant, antiparasitic, antimicrobial, anthelmintic, antibacterial, antiacne and acetylcholinesterase inhibitory activities [64,67,68,69,70]. A hand full of active compounds have been identified and isolated from *L. sericea* including triterpenes [66] and phloroglucinols [70]. As far as we know, procyanidins have not been identified from the plant previously and no nanoparticle synthesis reported.

Gold nanoparticles is one of the most widely used nanomaterials because of an array of interesting properties including the ease of its surface chemistry [40]. Among the importance of Au NPs is the ease of synthesis, characterization, surface modification, low toxicity, tunable surface plasmon resonance, biostability and biocompatibility [5,33,40,47,56,58]. The advantage that comes with these is the applications in biomedicals. Due to its small size, Au NPs can penetrate cells to interact with different molecules without causing damage making it the preferred candidate in drug delivery, cancer therapy among other applications [47,56].

Therefore, the aim of the present work was to identify procyanidins, a class of phytochemicals with unique structure from *Leucosidea sericea*, and use same for the fabrication and characterization of gold nanoparticles in order to understand the involvement of the functional groups in the gold nanoparticle formation. Biological activities of the intact fractions and nanoparticles were also evaluated through enzymatic and antioxidant studies.

## 2. Materials and Methods

### 2.1. General

Organic solvents, methanol (HPLC grade), ethanol, ethyl acetate and hexane, were supplied by Merck (Cape Town, WC, South Africa). All solvents used for extraction and column chromatography were general-purpose reagents. Silica gel 60 H (0.040–0.063 mm particle size supplied by Merck (Gauteng, Modderfontein, South Africa) and Sephadex LH-20 supplied by Sigma-Aldrich (Cape Town, WC, South Africa) were used as stationary phases, supported with a glass column of different diameters. The ^1^H, ^13^C and DEPT-135 Nuclear Magnetic Resonance spectra were run on a Bruker spectrometer operating at 400 (for H)/100 (for C) MHz. Polystyrene 96-well microtitre plates were obtained from Greiner bio-one GmbH (Frickenhausen, BY, Germany). Sodium tetrachloroaurate (III) dihydrate, iron (III) chloride hexahydrate, 2,4,6-Tris(2-pyridyl)-s-triazine, hydrochloric acid (HCl) and sodium chloride were procured from Sigma-Aldrich (Cape Town, WC, South Africa). N-Acetyl-L-cysteine and Folin–Ciocalteu’s phenol reagent were purchased from Boehringer Mannheim GmbH (Mannheim, BW, Germany). Glycine and phosphate buffered saline (PBS) were purchased from Lonza (Cape Town, WC, South Africa). BSA was procured from Miles Laboratories (Pittsburgh, PA, United States of America). Alpha-glucosidase (*Saccharomyces cerevisiae*), alpha-amylase (procaine pancreas), 3,5-dinitro salicylic acid (DNS), *p*-nitrophenyl-α-D-glucopyranoside (*p*-NPG), sodium carbonate (Na_2_CO_3_), sodium dihydrogen phosphate, and disodium hydrogen phosphate, Trolox (6-hydroxyl-2, 5, 7, 8- tetramethylchroman-2-carboxylic acid), 2,2-azino-bis (3-ethylbenzothiazoline-6-sulfonic acid) (ABTS) diammonium salt, potassium peroxodisulphate, gallic acid and vitamin C were purchased from Sigma-Aldrich (Cape Town, WC, South Africa). A polar star Omega microtitre plate reader (BMG Labtech, Ortenberg, BW, Germany) was used to monitor the characteristic peaks of the Au NPs. High-Resolution Transmission Electron Microscopy (FEI Tecnai G2 F20 S-Twin HRTEM, operated at 200 kV) was used to study the morphology of the Au NPs. A few drops of the gold suspension were dropped on a carbon-coated copper grid and allowed to dry completely at room temperature. An Oxford EDS system inside a Zeiss Auriga Field Emission Scanning Electron Microscope was used for elemental analysis. A few drops of the gold suspension were dropped on a Mica glass substrate and allowed to dry. The crystal structures of the samples were determined by X-ray diffraction (XRD; X-ray diffraction Model Bruker AXS D8 advance) with radiation at λkCuka1 = 1.5406 Å. Dynamic Light Scattering (DLS) analysis was done using a Malvern Zetasizer Instrument (Malvern Ltd., United Kingdom) at 25 °C and a 90° angle. Zetasizer software version 7.11 was used to analyze the data. The absorbance readings of biological activities were measured at 540 nm using Multiplate Reader (Multiska Thermo scientific, version 1.00.40)

### 2.2. Extraction and Purification of F1 and F2 from LSTE

The powder of the aerial parts of *Leucosidea sericea* (107.40 g) was extracted with 50% aqueous-ethanol (1.2 L). The extraction process was conducted for 72 hr under room temperature with occasional manual agitation. The filtrate after concentration in vacuo afforded 36.20 g (33.71%). About 35.30 g was dissolved in 0.5 L of water and successively partitioned with 0.6 L hexane, dichloromethane (1.0 L) and 1.0 L of ethyl acetate. Ethyl acetate fraction (2.00 g) was applied to the silica gel column and eluted with a mixture of hexane-DCM/DCM-EtOAc/EtOAc-MeOH of increasing polarity. Collected fractions were pooled together according to their profiles (on the TLC) to afford 6 major fractions coded as LST1–6. Fraction LST 4 (79.2 mg) was further subjected to the Sephadex column (LH-20) using 80% toluene-ethanol and resulted in the isolation of one single spot (62.3 mg) and named F1. The more polar fraction LST 6 was subjected to Sephadex column using 20% aqueous ethanol and resulted in one single spot (18.0 mg), more polar than the previous one and coded as F2. The analysis was achieved by ^1^H, ^13^C, DEPT-135 NMR (Supplementary data, Figures S1–S6) and LC–MS.

### 2.3. Liquid Chromatography–Mass Spectrometry (LC–MS) Analysis

A Waters Synapt G2 quadrupole time-of-flight (QTOF) mass spectrometer (MS) connected to a Waters Acquity ultra-performance liquid chromatography (UPLC; Waters, Milford, MA, United States of America) was used for high-resolution UPLC–MS analysis. The method and conditions described in reference [71]

### 2.4. Green Synthesis of Gold Nanoparticles

The Au NPs was synthesized by dissolving 0.01 g of the total extract, F1 or F2, in 1 mL of deionized Milli-Q water and vortexed for 10 min giving a clear yellowish solution. This solution was emptied into a pre-heated 1.0 mM sodium chloroaurate solution at about 90–100 °C. An instantaneous change of color from light yellow to reddish indicated successful synthesis of Au NPs. Source of heat was turned off, while reaction mixture continued to stir for additional 60 s. UV-Vis, particle size and zeta potential measurements were done upon cooling to room temperature.

### 2.5. Stability Study

The method description of [72] was adopted. Slight changes were only in the monitoring period, which was 0, 12 and 24 h. The stability was evaluated as soon as the solutions were mixed with the nanoparticles and after incubation at 37 °C.

### 2.6. Dilution Study

To get the Au NPs in the powdered form, 100 µL of the sample was freeze-dried after several washing and centrifuge steps. Of the synthesized Au NPs, 200 µL at different concentrations (100, 80, 60, 40, 20 and 10 µg/mL) were prepared using deionized water in a 96-well microtitre plate and the absorbance was taken by using a polar star Omega microtitre plate reader. From the absorbance curve, the intensity was plotted against different concentrations to examine the relationship and stability of the nanoparticles.

### 2.7. In-Vitro Methods Employed in Antidiabetic Studies

#### 2.7.1. Alpha-Amylase Inhibitory Activity

The alpha-amylase inhibitory activity of the *Leucosidea sericea* total extract (LSTE), F1, F2 and their corresponding gold nanoparticles were carried out according to the standard method with slight modifications [73]. In a 96-well plate, 50 μL phosphate buffer (100 mM, pH = 6.9) was added followed by 20 μL alpha-amylase (2 U/mL) and 20 μL of varying concentrations of above solutions (200, 100, 50, 25 and 12.5 µg/mL) were pre-incubated at 37 °C for 20 min. Thereafter, 20 μL of 1% soluble starch (100 mM phosphate buffer pH 6.9) was added as a substrate and incubated again at 37 °C for 30 min. Then, 100 μL of the DNS color reagent was added and boiled for 10 min. The absorbance of the resulting mixture was measured at 540 nm using a plate reader (Multiskan Thermo scientific, version 1.00.40). Acarbose at various concentrations (0.1–0.5 mg/mL) was used as a standard. Without test (solutions of extract, F1, F2 and nanoparticles) were set up in parallel as control and each experiment was performed in triplicate. The results were expressed as percentage inhibition, which was calculated using the formula below;

Inhibitory activity (%) = (1 − A/B) × 100

where A is the absorbance in the presence of test substance and B is the absorbance of control.

#### 2.7.2. Alpha-Glucosidase Inhibitory Activity

In addition to the procedure of [74], all other sample preparations are the same as in Section 2.7.1.

### 2.8. Antioxidant Activity

#### 2.8.1. Ferric Reducing Antioxidant Power (FRAP) Assay

FRAP assay was done on LSTE, F1 and F2 and their corresponding Au NPs. The method of [75] was followed with a slight adjustment. Absorbance was measured at 593 nm and the results were expressed as μM ascorbic acid equivalents per milligram of dry weight (μM AAE/g DW) of the samples.

#### 2.8.2. Total Phenolic Content

This evaluation of amount phenolics in LSTE, F1 and F2, as well as their corresponding Au NPs, was done by the method of Salar and colleagues [76] with slight modification. The plate was read at 593 nm and the results expressed as gallic acid equivalent.

#### 2.8.3. 2′-Azino-Bis-3-Ethylbenzotiazolin-6- Sulfonic Acid (ABTS) Assay

The ABTS assay was conducted adopting a combined procedure reported in [77,78].

## 3. Results and Discussion

### 3.1. The L. sericea Constituents and Gold Nanoparticle Formation

Chromatographic manipulation of the LSTE using different chromatographic techniques resulted in the purification of two fractions (F1 and F2) with potent Au NPs formation. The total extract and purified fractions were investigated for their chemical constituents using different techniques. Although the NMR spectroscopy is the most important technique for structural elucidation in natural products, the LC–MS techniques are the best and fastest method in case of the complicated mixture and to unveil the nature of the chemical constituents.

**Figure 1:**
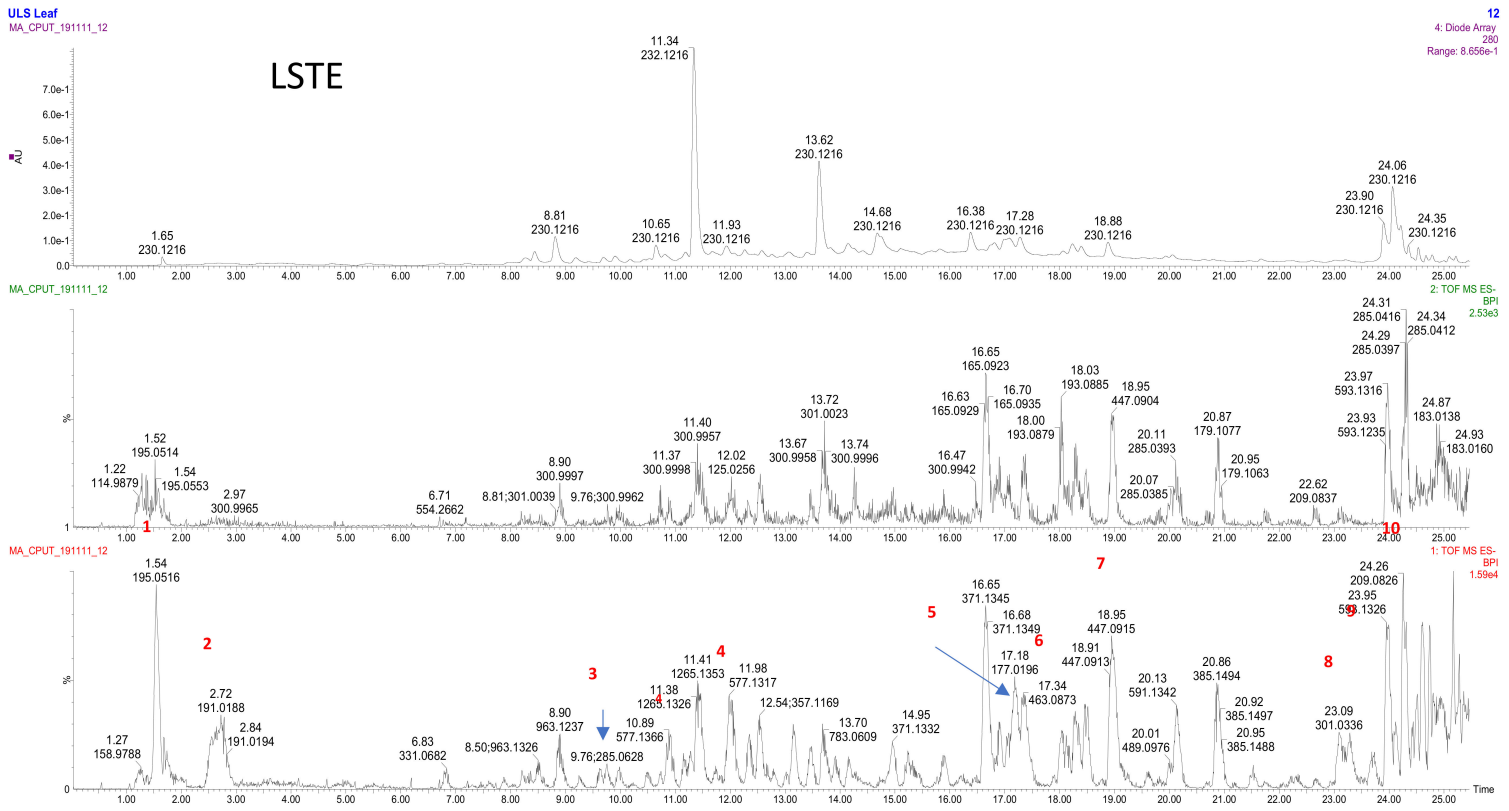
LC–MS profile of the *L. sericea* aerial part extract. 1: ferulic acid; 2: quinic acid; 3: luteolin 4: procynanidin dimer; 5: caffeic acid; 6: hyperoside, 7: kaempferol galactoside; 8: quercetin; 9: kaempferol rutinoside; 10: hydroxyferulic acid.

**Scheme 1:**
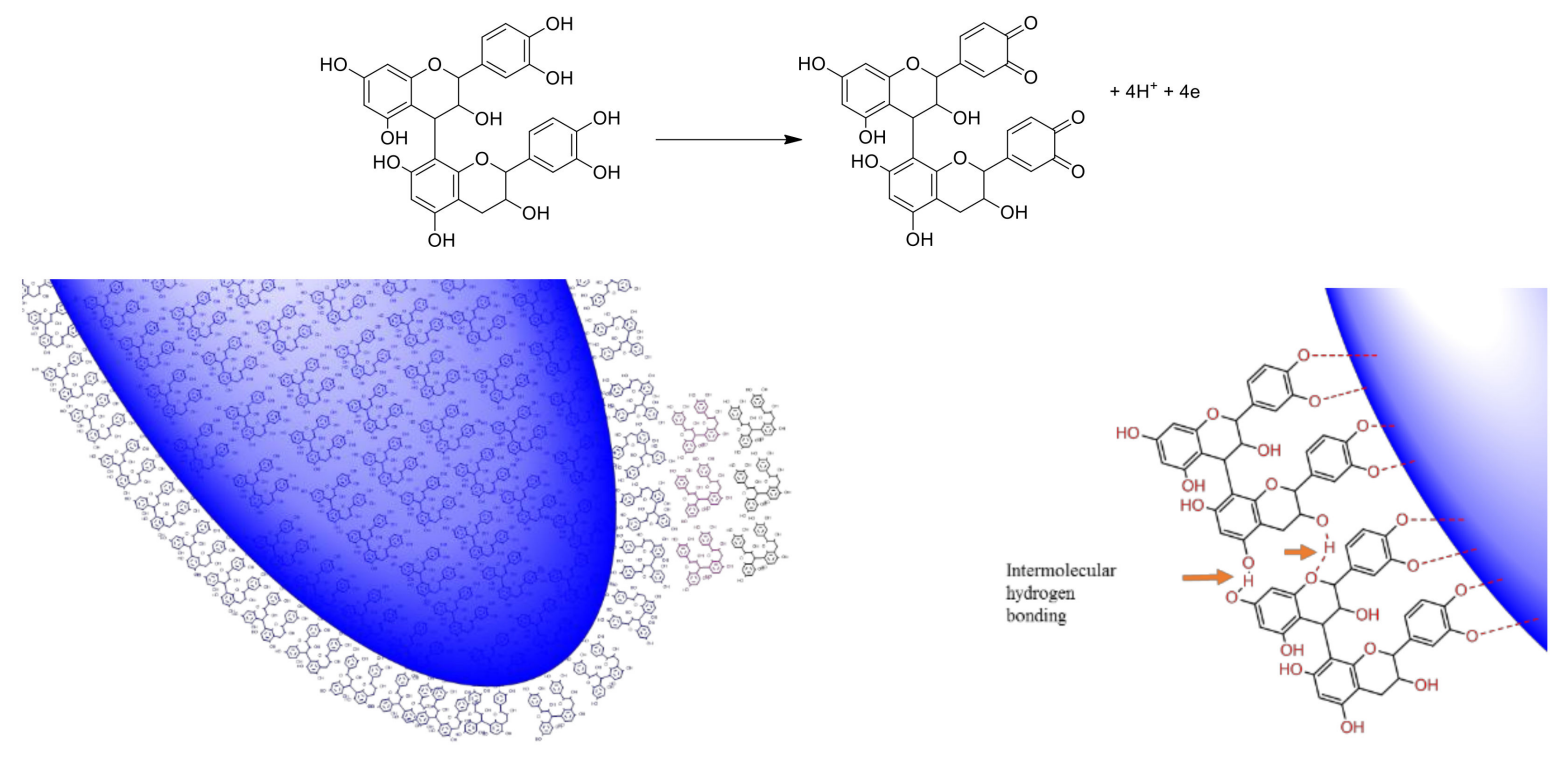
The proposed reduction of gold salt and encapsulation of the hybrid gold NPs in the F1 and F2 matrix. In addition to the chelating power of the oxygen atom to the metal surface, the intermolecular hydrogen bonding between procyanidins can also make double and triple capping shells and contributing to the stability of the NPs. The reduction power of the phenolic structures with *ortho*-OH, can be facilitated by the formation of stable *ortho*-quinone structure.

The LC–MS analysis of the LSTE (Figure 1) showed wide range of *m*/*z* (M-H) ions, which indicated the presence of different phenolic compounds including phenolic acids (ferulic (*m*/*z* 195/R_t_ 1.52 min)/quinic (191/2.72)/hydroxyferulic (209/24.26) and caffeic (177/17.18) acids); flavonones ((luteolin (285/9.76), quercetin (301/23.09), hyperoside (463/17.34), kaempferol galactoside (447/18.95) and kaempferol rutinoside (593/23.95)) and procyanidin dimers (577/10.89, 11.98; B type). These types of compounds have a strong ability to donate electrons and are playing a key role in the oxidation–reduction process; the removal of the electrons results in a stable quinonoidal structure (Scheme 1).

**Figure 2:**
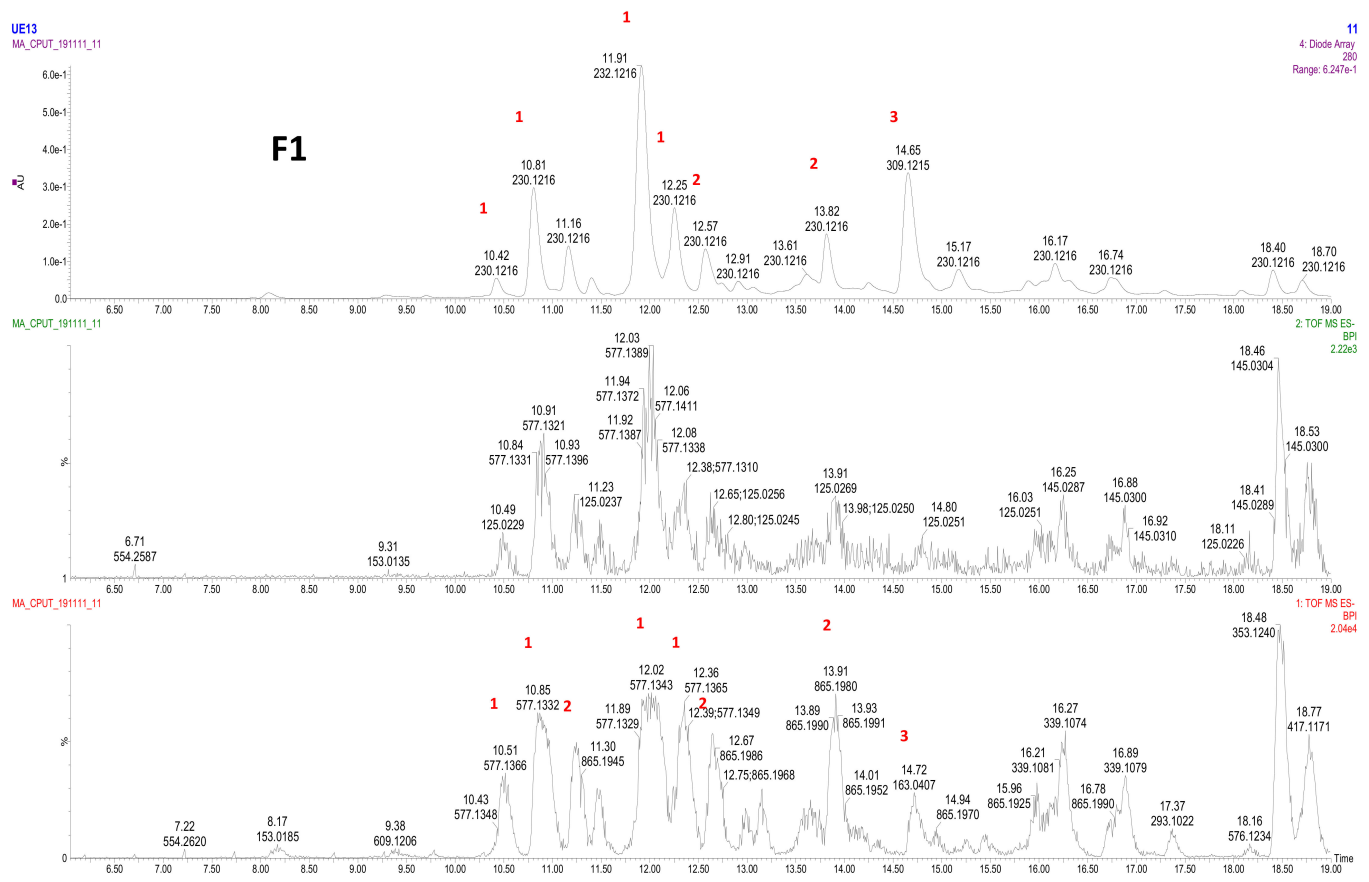
LC–MS profile of F1. 1: procyanidin dimer (type B); 2: procyanidin trimer (type B); 3: coumaric acid.

Attempted purification of fraction (F1) showed both dimers (R_t_ 10.51; 10.85; 12.02; 12.36) and trimers (R_t_: 11.30; 12.67; 13.91; 14.94; 15.96; 16.78), however, the relative quantity of dimers was higher than trimers (Figure 2).

**Figure 3:**
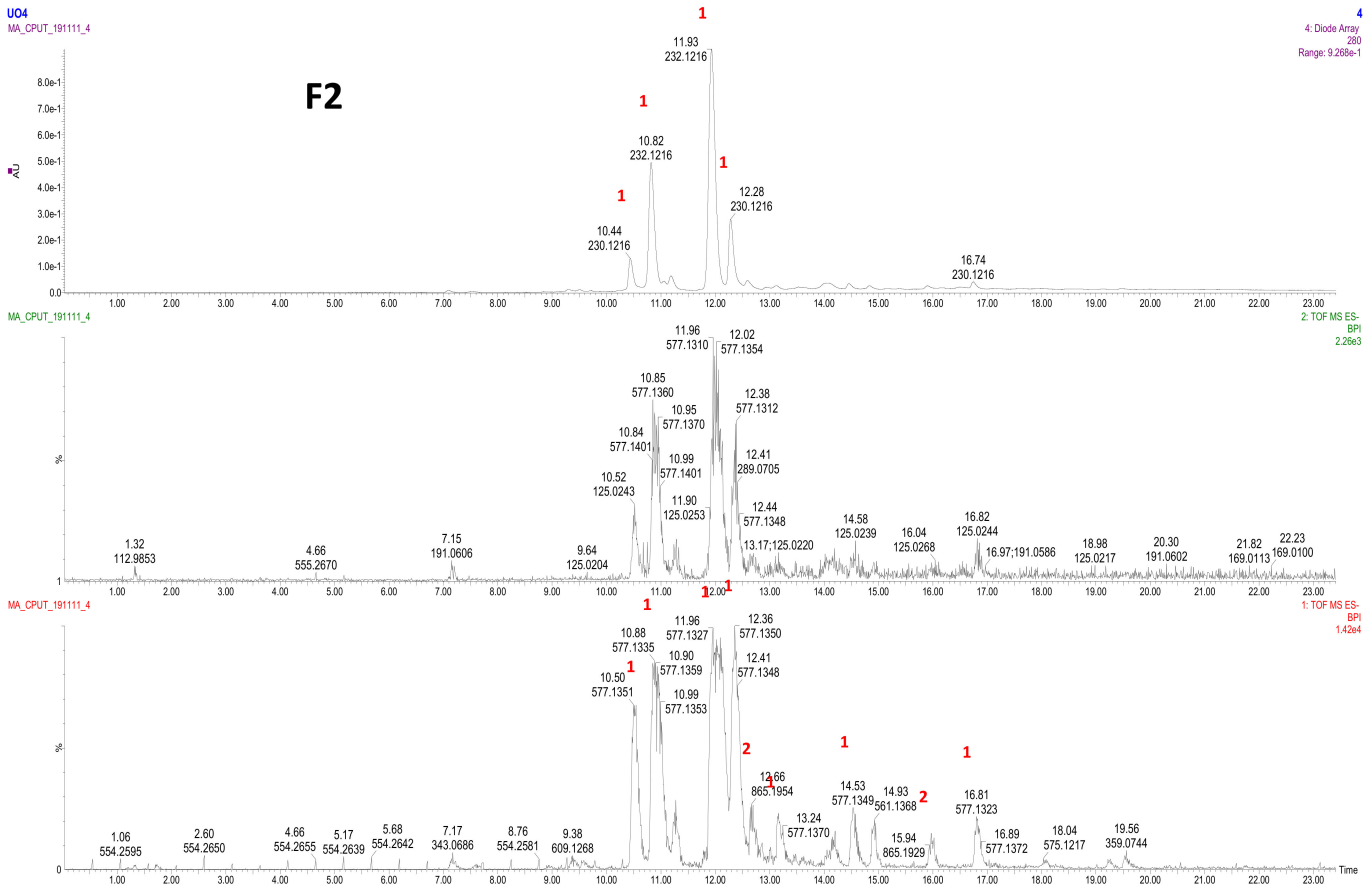
LC–MS profile of F2. 1: procyanidin dimer (type B), 2: procyanidin trimer (type B).

Further purification of fraction LSTE4 produces a single spot, which initially was believed to be a pure compound. However, the NMR (^1^H and ^13^C NMR), and LC–MS analysis showed an isomeric mixture. The LC–MS of F2 (Figure 3) showed procyanidin dimer peaks B-type (*m*/*z* 577) at R_t_ of 10.50; 10.88; 11.96; 12.36; 13.24; 14.53 and 16.81; the first four peaks were major while the others were minor. The trimers (B-type; *m*/*z* 865) were also detected in minute quantities at 12.66 and 15.94 (Figure 3).

LSTE, F1 and F2 were then used to successfully form Au NPs. The polyphenolic nature of these fractions makes them hydrophilic, a requirement for green synthesis. The reducing and capping ability of polyphenolics has been associated with the presence of electronegative atoms [79]. The lone pairs of electrons on oxygen further provide a suitable chelation site for binding with gold. The arrangement of certain functional groups in the structure of polyphenolics is also of great significance in their reactivity. Hydroxyl groups at adjacent positions may offer two or more binding sites, which may enhance the easy reduction of gold and subsequent capping of the particles that are formed. Oxidation reactions are common to phenolic compounds where the hydroxyl groups at ortho and para positions convert to carbonyl groups (*ortho* and/or *para* quinones). In the case of the procyanidins, the oxidation will occur at ortho positions (Scheme 1) when 4 and 6 electrons are lost for the dimer and trimer respectively. Subsequent interaction of the particles with the molecule probably brought about weak forces of attraction and encapsulation in the F1 and F2 matrix (Scheme 1). As would be discussed later, the particles are covered with negative charges providing stability and confirming the negative zeta potential values as measured by zetasizer.

### 3.2. UV-Visible Spectroscopic Analysis

**Figure 4:**
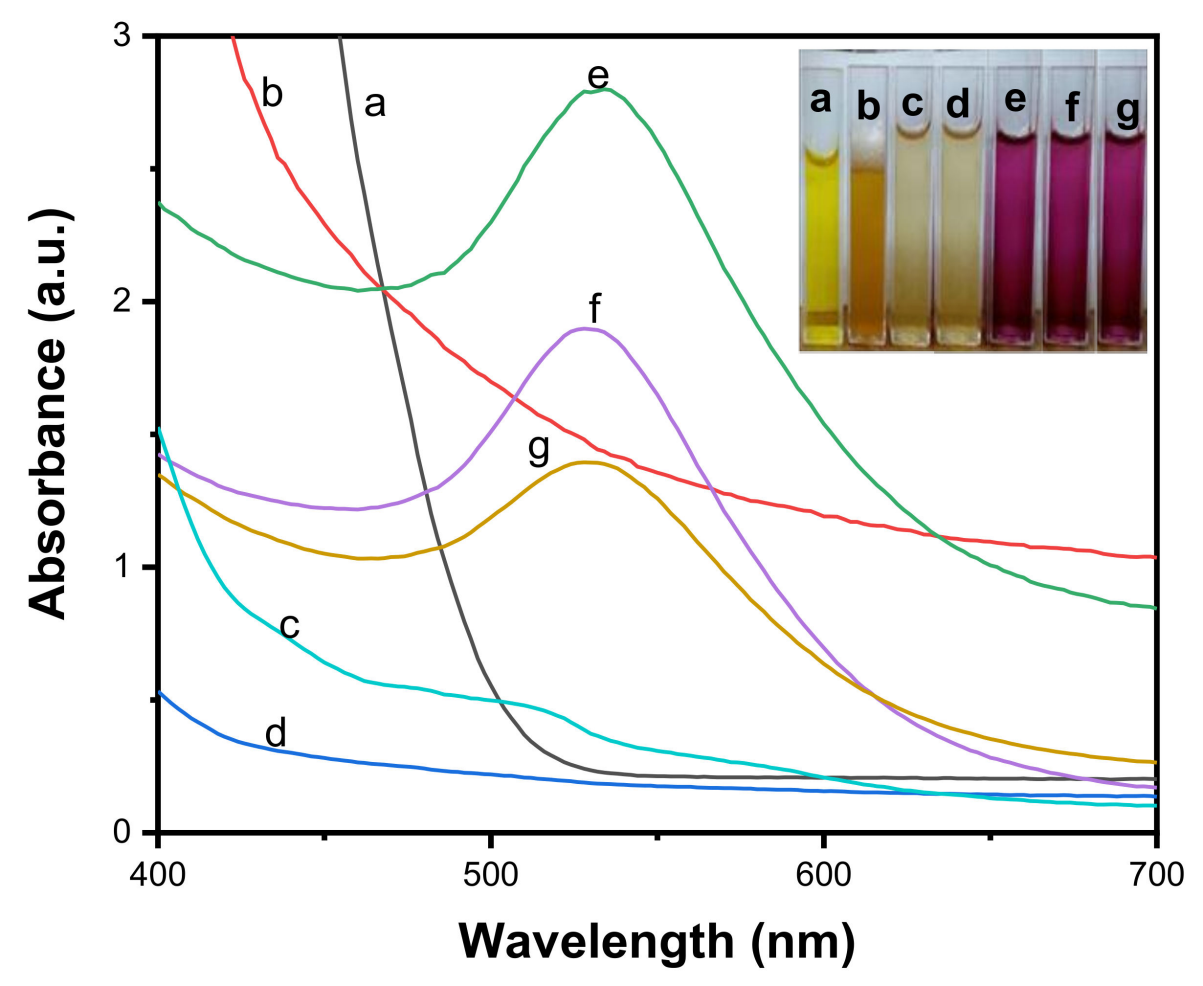
Absorption spectra of (**a**) gold solution, (**b**) aqueous solution of the *Leucosidea sericea* total extract (LSTE), aqueous solution of (**c**) F1 and (**d**) F2 and the corresponding Au NPs of (**e**) LSTE, (**f**) F1 and (**g**) F2.

The absorbance spectra of (a) gold salt solution, an aqueous solution of; (b) LSTE, (c) F1, (d) F2 and the Au NPs prepared from (e) LSTE, (f) F1 and (g) F2 are shown in Figure 4. Au NPs in general, are known to have surface plasmon resonance (SPR) at around 530 nm [71]. The Au NPs, prepared from LSTE, F12 and F2 revealved SPR of 528–532 nm, confirming successful Au NPs fabrication. Before the reduction process, no absorption peaks were observed in such a region. The color of LSTE, F1 and F2 were also yellowish, ranging from deep yellow to pale yellow (inset in Figure 4). These colors changed to ruby red when the gold salt was introduced, which served as a visual confirmation of the gold nanoparticle fabrication. The phytochemicals in LSTE, F1 and F2 therefore, acted as reducing and subsequently as capping agents in the respective newly formed Au NPs, preventing aggregation of the particles. It was observed from Figure 4 that the SPR of both F1 and F2 Au NPs were closely related. This is probably because of the similar constituents as discussed above. Hence, dimers and trimers of procyanidins were clearly involved in the successful fabrication of F1 and F2 Au NPs. Furthermore, since no external stabilizers were employed, it could also be concluded that the same constituents were responsible for the stabilization of the nanoparticles (see Scheme 1). Many investigations concluded that polyphenols, which are abundant in plants, are associated with the reduction of metal salts when combined with plant extracts [47,48]. This may also explain why F1 and F2 (which contain similar functionality) were not only able to reduce gold to its zero-valence state but also formed Au NPs with very similar characteristics to that of the LSTE.

### 3.3. High-Resolution Transmission Electron Microscopy (HR-TEM)

**Figure 5:**
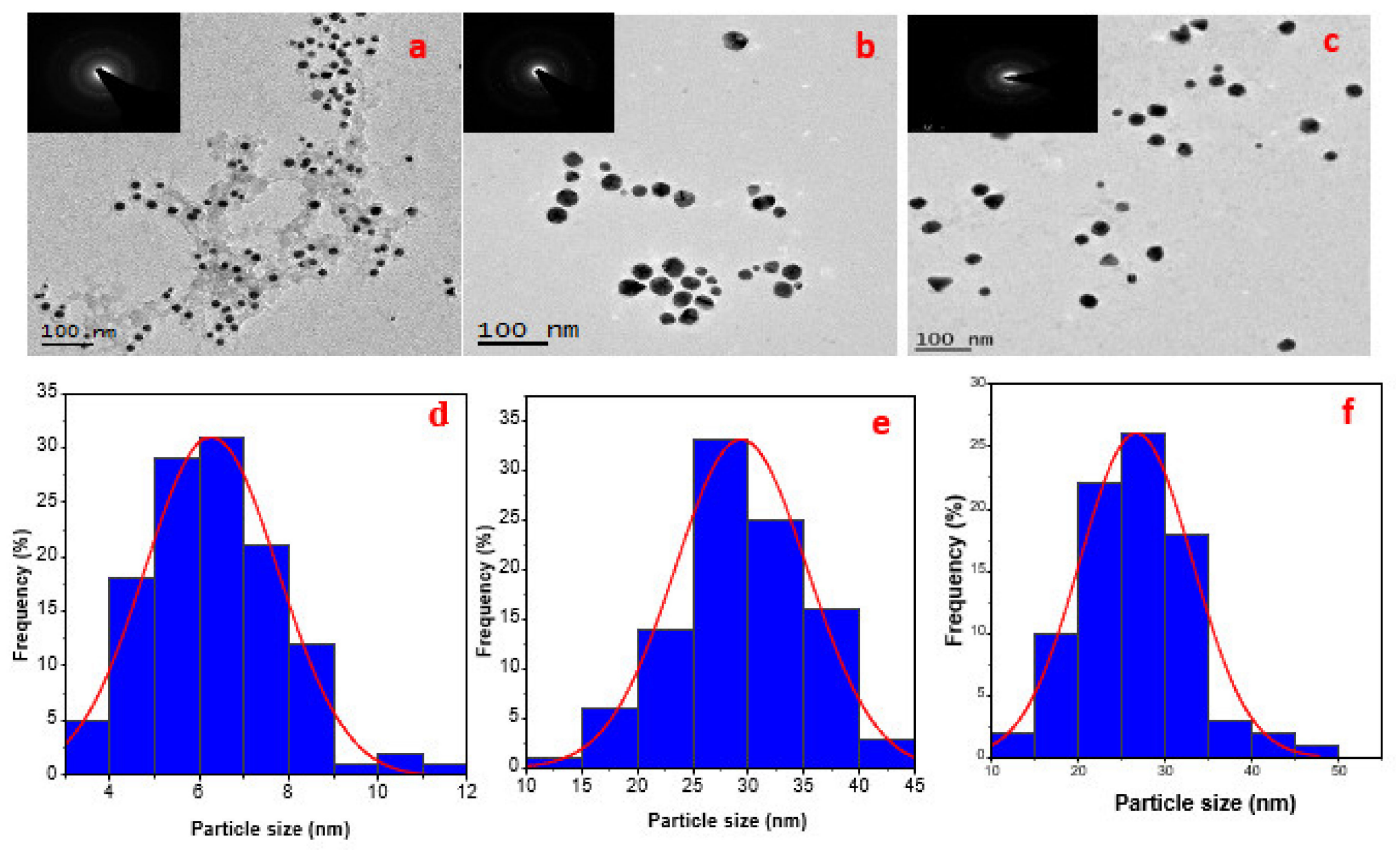
HRTEM images for LSTE, F1 and F2 Au NPs are represented as (**a**–**c**) and the corresponding particle size distributions as (**d**–**f**) respectively. The inset in a, b and c, showing the bright rings are the SAED of the respective TEM images.

HRTEM provides information regarding morphology, crystallinity and size of nanoparticles. Figure 5a–f displayed the HRTEM results and the evaluation of particle size of LSTE, F1 and F2 Au NPs. The mean diameters of the particles were 6.3 ± 2, 24.4 ± 5 and 22.4 ± 5 nm respectively. Figure 5 shows that Au NPs were predominantly monodispersed with a spherical shape. Thick coatings could also be observed surrounding the particles, which might be extra stabilizing support from other phytochemicals in the leaf extract serving as capping agents to the hybrid nanoparticles formed. Coatings up to 5.83 nm in width surrounded particles in LSTE Au NPs and about 2.50 nm for F1 Au NPs (Figure 5b). Nevertheless, the F2 Au NPs seeds had a layer of 1.97 nm thick, mostly on the spherical particles. The approximate similar size of Au NPs conjugated with F1 and F2 can be explained in terms of the similarity in the structures and capping layers packing of F1 and F2, which (both) contain procyanidin dimers (F2) or a mixture of dimers and trimers (F1). On the other hand, the smaller size obtained by the total extract may indicate the strength of the reducing agents. It could also be due to the presence of smaller sized molecules that have the ability to perfectly shield the Au NPs. Similar coatings have been observed previously having a width between 2 and 3 nm and it was concluded that it prevents the gold nanoparticles from agglomeration [80]. Smaller particles are predominantly round-shaped, others are polygonals including traingular and some five sided shapes. On the other hand, particles with relatively larger sizes were mostly hexagonal and truncated triangular. This combination of shapes is characteristic of Au NPs and it has been associated with the different chemical constituents forming the Au NPs [81,82]. Furthermore, it is interesting to note that the mixture of shapes is common to the HRTEM results of the Au NPs but much more pronounced for F1 and F2 Au NPs. This further serves as evidence of the involvemnet of similar functional groups in forming the two particles. The inset in Figure 5a–c shows the selected area electron diffraction (SAED) pattern of LSTE, F1 and F2 Au NPs respectively. The bright circular rings indicate that the particles are polycrystalline, and can be linked to the (111), (200), (220) and (311) planes of a face-centered (FCC) structure of gold [83].

**Figure 6:**
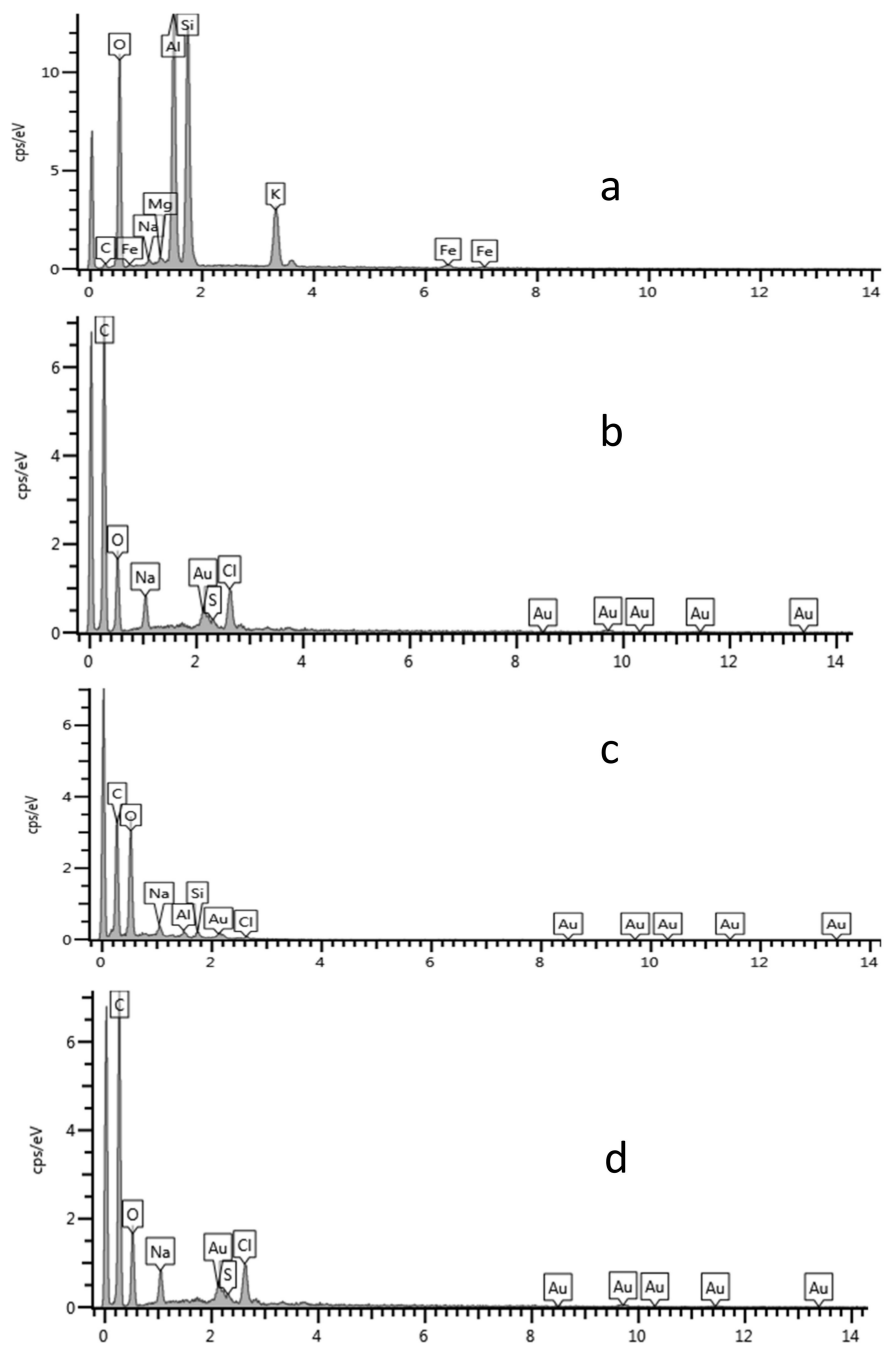
EDS spectra of (**a**) blank, (**b**) LSTE Au NPs, (**c**) F1 Au NPs and (**d**) F2 Au NPs.

The EDS spectra of the blank/glass substrate (Figure 6a) and the Au NPs (Figure 6b–d) confirmed the presence of Au in the solution. Other elemental peaks such as C, O and Na (Figure 6a–d) were likely from the sample preparation/glass substrate used to prepare the samples for the analysis. The small sulfur peak (Figure 6b) could be a contaminant while Cl and perhaps some of the Na were possibly from the sodium chloroaurate solution used as a precursor.

### 3.4. X-Ray Diffraction (XRD) Analysis

**Figure 7:**
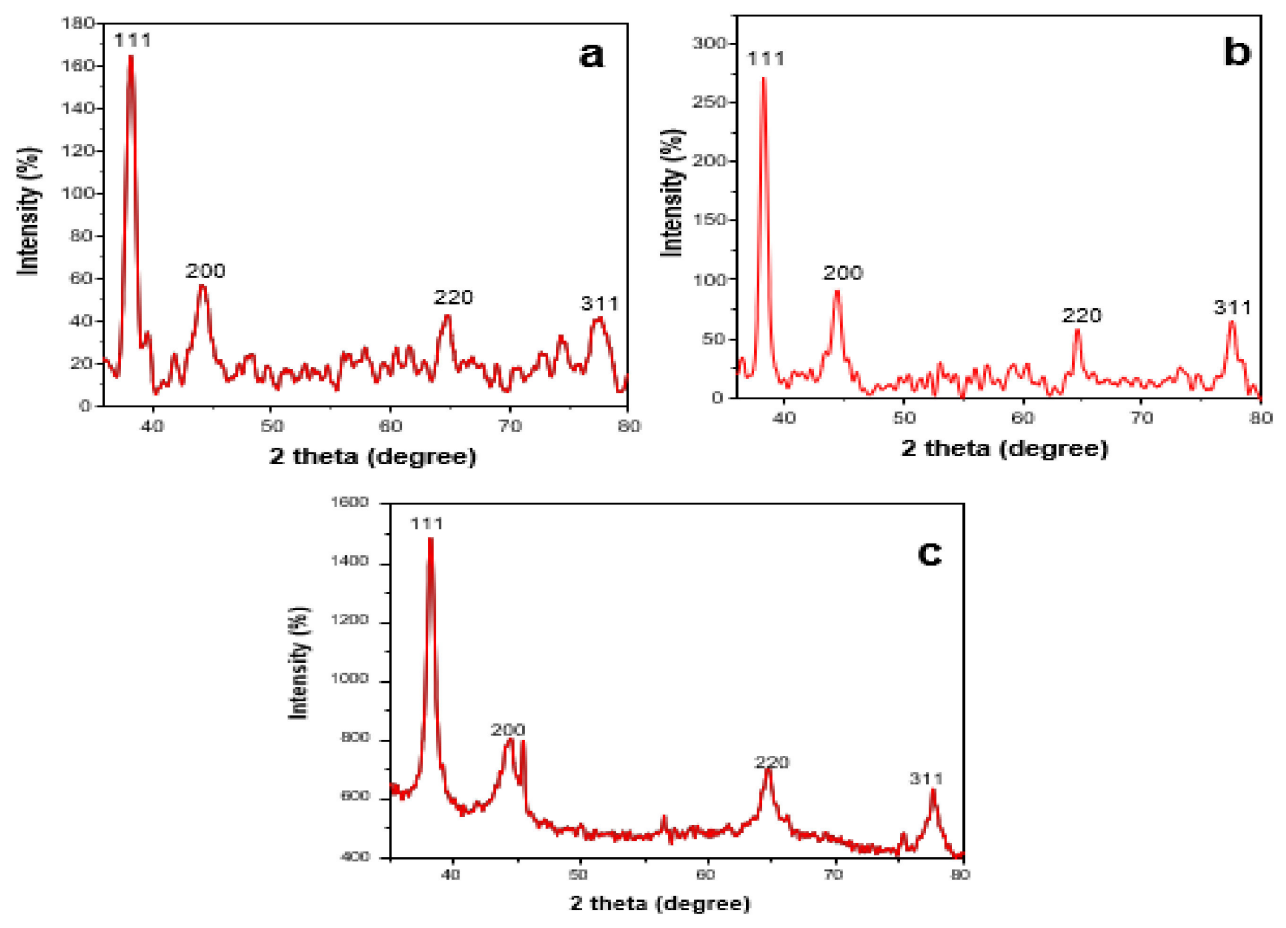
XRD patterns of the (**a**) LSTE Au NPs, (**b**) F1 Au NPs and (**c**) F2 Au NPs.

Figure 7 depicts XRD patterns of Au NPs of LSTE, F1 and F2. Four distinct peaks (Figure 7a–c) were observed at 2 theta degree values equal to 38.2, 44.4, 64.6 and 77.5. These corresponds to the (111), (200), (220) and (311) planes respectively of the FCC gold lattice [74], in agreement with that of the pure crystalline gold structure published by XRD Joint Committee on Powder Diffraction Standards (file nos. 04-0784). XRD data were further corroborated by the bright circular rings observed in the SAED patterns, as shown earlier (Figure 5).

### 3.5. Dynamic Light Scattering (DLS) Measurement of Au NPs

**Figure 8:**
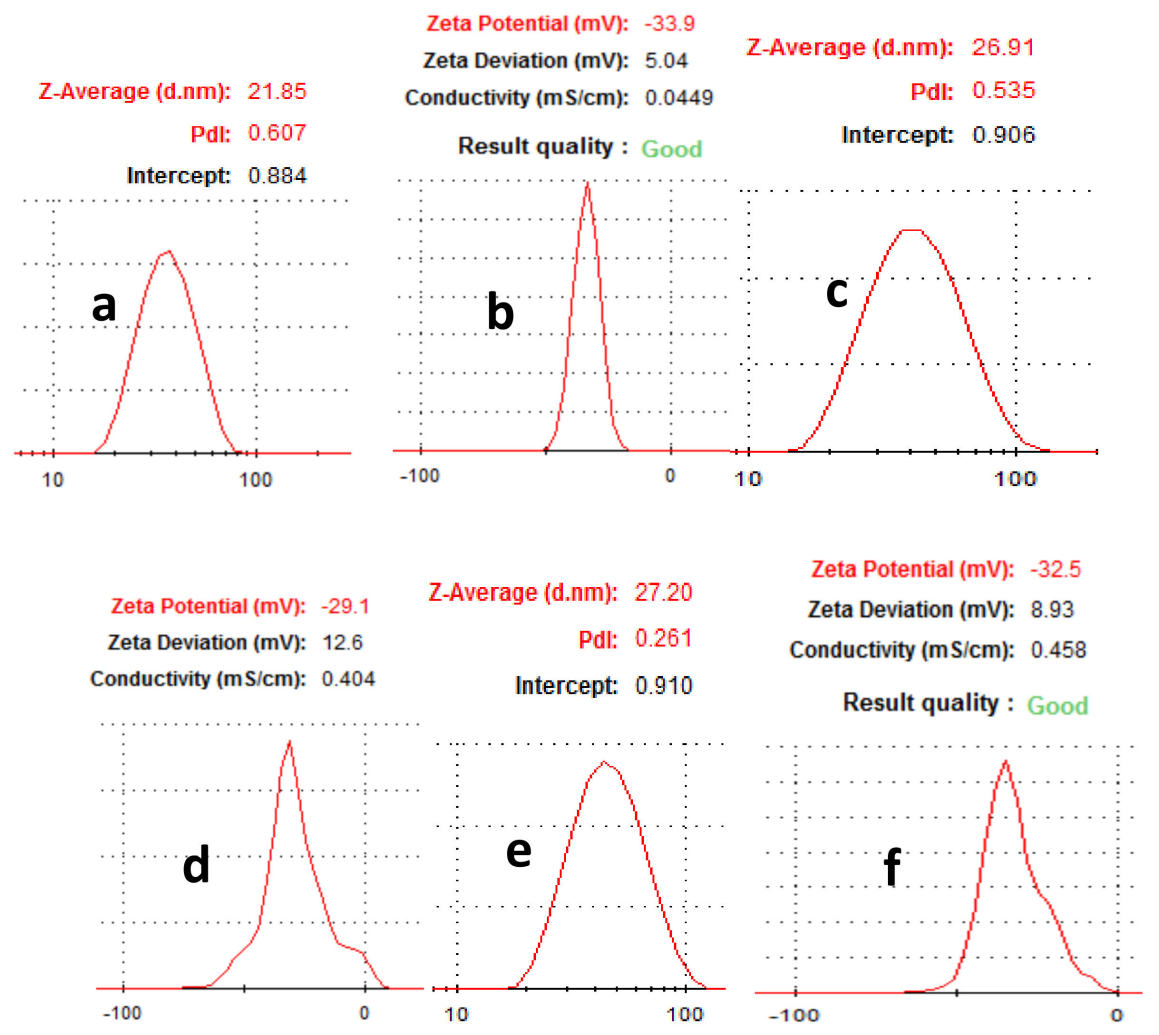
Hydrodynamic size of (**a**) LSTE, (**c**) F1, (**e**) F2 and zeta potential of (**b**) LSTE, (**d**) F1 and (**f**) F2 Au NPs measured using Dynamic Light Scattering (DLS).

Among other important factors to be considered before the application of nanoparticles is the phase behavior. Light scattering is one of the techniques to understand this phenomenon. DLS, a non-intrusive technique, was employed to measure the size and zeta potential of the Au NPs. The average size of LSTE, F1 and F2 Au NPs was found to be 21.85, 26.91 and 27.20 nm respectively (Figure 8). Size characterization of nanoparticles is of immense importance in nanomedicine. However, because of the physical properties that are measured, and the weighted averages determined in each case, the DLS measurement for size is often not the same as those measured by electron microscopy [84]. The size variation between DLS and TEM could therefore be due to the sampling volume used during analysis.

The zeta potentials (ZP) taken immediately after particle formation are displayed in Table 1. Values of −33.59, −32.5 and −29.1 mV (Table 1) were obtained for the LSTE-, F1- and F2 Au NPs respectively. ZP is related to the surface charge of nanoparticles, and particles with ZP values -30 mV and below are taken to be mostly covered with negatively charged ions. The opposite is true for positive ZP values. The extent of ZP is therefore, a subject of repulsion and attraction that has been used to estimate how long the nanoparticle dispersions would remain stable. The negative zeta-potential values obtained for the Au NPs fall in the range that is typical of stable colloidal dispersions [72]. The ZP measurement for the nanomaterials was repeated on the seventh day through to the fourteenth day showing only slight changes. From the DLS measurement, a slightly higher value of ZP for LSTE Au NPs could be due to the synergistic effect of other compounds present, probably providing enhanced capping ability. Both the hydrodynamic sizes and ZP values for F1 and F2 can be observed to be approximately the same and therefore can be explained by the similarity of phytochemicals involved. This was also supported by their HRTEM results, where the average particle sizes were 24.4 and 22.4 nm for F1- and F2 Au NPs respectively. Interestingly, their ZP values equally indicated that the particles are stable to a similar extent, further confirming the presence of similar capping agents. Significant changes were not noticed in the ZP values for the given days, thereby suggesting no agglomeration as they repelled each other. Therefore, the particles were quite stable.

### 3.6. In Vitro Stability Study

**Figure 9:**
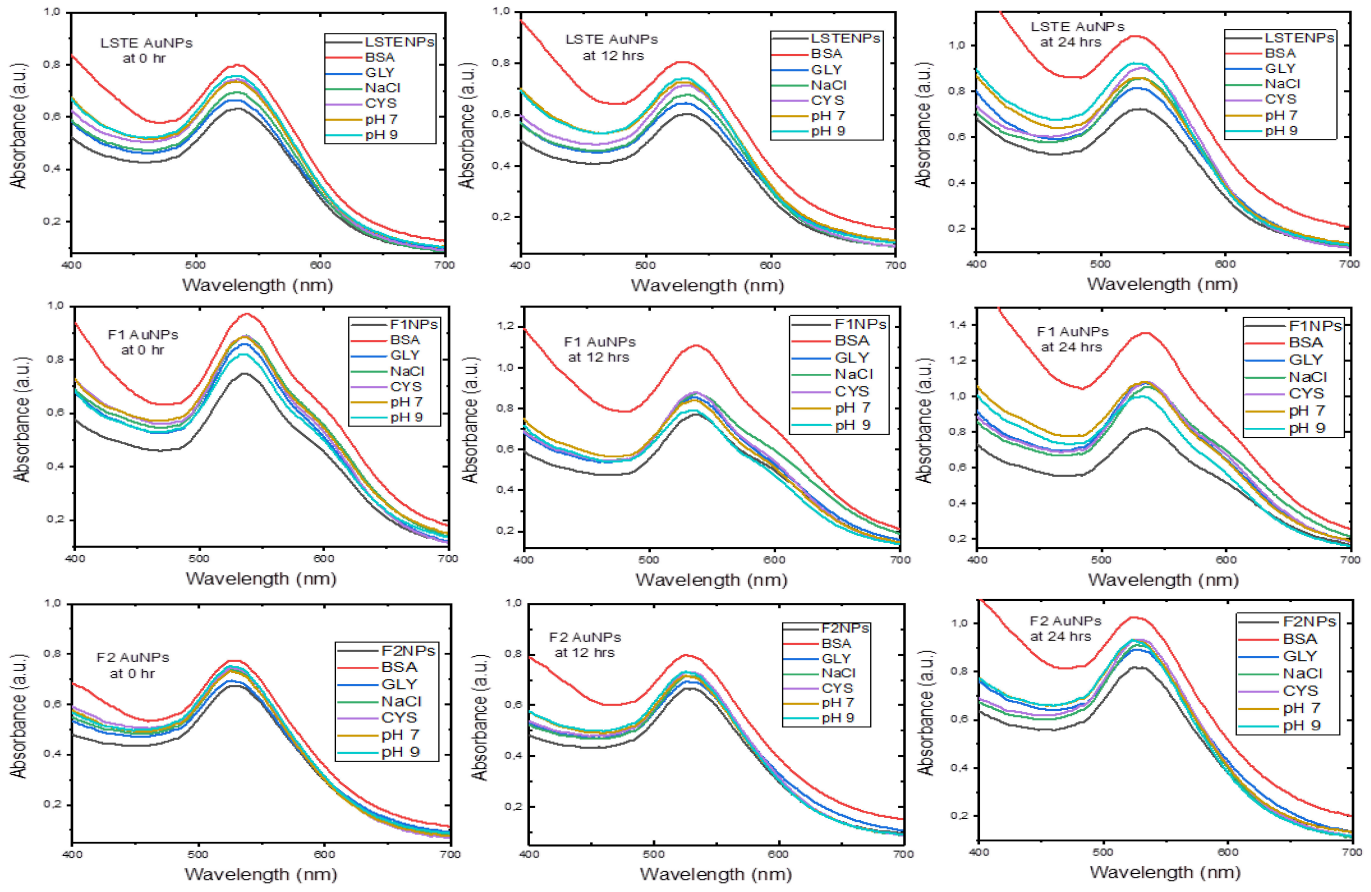
Stability of gold nanoparticles at given time intervals.

The effectiveness of nanoparticles in medical applications depends on its stability in biological solutions for a reasonable period. Figure 9 displayed the evaluation of stability when 0.5% bovine serum albumin (BSA), glycine (GLY), sodium chloride (NaCl), cysteine (CYS) and phosphate buffers at pH 7 and 9 were mixed with LSTE, F1 and F2 particles [85]. These were carried out before and after incubation at 37 °C. Even though the pH 9 solution is higher than the pH of human body fluids, it was considered to get additional information about whether nanoparticles are still stable at such a high pH. The results showed that the SPR remained the same in all the formulations. However, a noticeable shift by BSA especially from the 12th h is a behavior of certain proteins. BSA has been reported to have preference of binding to negatively charged surfaces [86]. This has been ascribed to the presence of over 50 surface lysine groups, which enable easy electrostatic interactions with negative surfaces. Consequently, the negative ZP values of our particles implies that the surfaces were largely covered by negative ions, hence more interaction with BSA. Overall, the shifts of less than 1.0 nm in all solutions was minimal, the λ_max_ remained intact thereby affirming the stability of the particles in the solutions at the tested period.

### 3.7. Dilution Study

**Figure 10:**
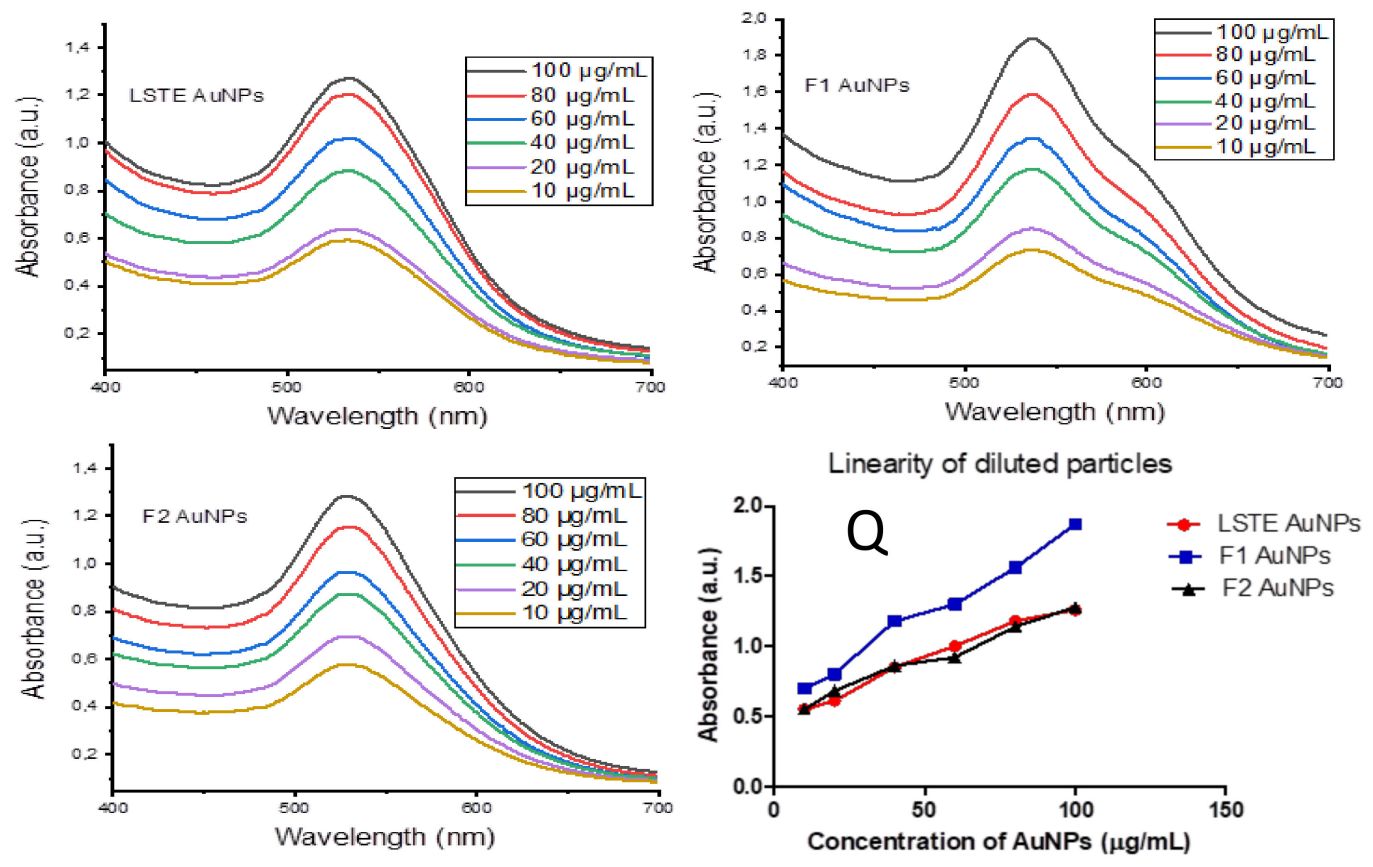
Surface plasmon resonance wavelength (λ_max_) of diluted concentrations of LSTE-, F1- and F2- Au NPs, while (**Q**) represents a plot of absorbance against various concentrations, showing the linear proportionality of intensity with increasing concentration. R2 = 0.9455 (LSTE Au NPs), R2 = 0.9908 (F1 Au NPs) and R2 = 0.9848 (F2 Au NPs).

Some biomedical applications require different concentrations of gold nanoparticles. Figure 10 shows the absorbance of the hybrid (a) LSTE-, (b) F1- and (c) F2 Au NPs at different concentrations. To confirm that dilution of nanoparticles into different concentrations does not affect their stability and does not alter their physical and chemical properties in vivo, a dilution study was carried out at different concentrations of the nanoparticles (100, 80, 60, 40, 20 and 10 µg/mL), and UV-Vis spectroscopy (350–850 nm) was recorded for each concentration. From the UV-Vis spectra, the SPR wavelength had the same value for all the solutions of a given particle. This means that the dilution did not have an effect on the properties of LSTE-, F1- and F2-Au NPs, and the nanoparticles remained stable. The relationship between various concentrations of the nanoparticles and the absorbance was further examined and found to be linear. Figure 10Q shows that the intensity of absorption had linear dependence on the concentration of respective nanoparticles. This is additional evidence that the particles were stable and that they did not agglomerate even as the concentrations change.

### 3.8. In-Vitro Antidiabetic Studies

Since T2DM is associated with neurological and cardiovascular complications as a result of metabolic disorders emanating from hyperglycemia, the most critical remedy is to keep the blood sugar within the normal range. Two special enzymes, alpha-glucosidase and -amylase have good records of breaking down the long-chains of carbohydrates thereby enhancing the breakup of the glucose unit from the disaccharide. Therefore, the inhibition of these enzymes has been among the popular therapies for T2DM [87]. The antidiabetic properties of plant extracts have been reported before [88]. In this study, *Leucosidea sericea* leaves extract, the fractions (F1 and F2) and gold nanoparticles biosynthesized from them were studied and their potential as antidiabetic and antioxidant agents evaluated.

For the enzymatic studies, strong inhibitory activities were displayed by the constituents of *Leucosidea sericea* as well as the fabricated Au NPs (Table 2). F1 and F2 are fractions from the extract and demonstrated high alpha-glucosidase activity. IC_50_ values of 8.1, 7.3 and 7.1 µg/mL were obtained for LSTE, F1 and F2 respectively. The alpha-amylase inhibition resulted in 3.5 µg/mL for LSTE and 18.9 µg/mL for F2. F1 did not show any activity at the tested concentrations. In general, the results showed that LSTE, F1, F2 and their Au NPs had the potential to be used as antidiabetic agents. Pure isolated phytochemicals have demonstrated activities against alpha-glucosidase in previous studies [47,48]. Recently, Etsassala et al. [89] reported a strong antidiabetic activity of ten abietane diterpenes. In their study, the antidiabetic activity was due to the type and position of different functional groups in the compounds. Additionally, some compounds displayed either alpha-glucosidase or alpha-amylase activity and not both. For instance, 11,12-dehydroursolic acid lactone displayed moderate alpha-glucosidase activity but showed no activity for amylase [89]. Although, both fractions (F1 and F2) have similar structures, F1 did not show activity against alpha-amylase compared to F2, this may be due to the antagonistic effect of one of the trimers, and this effect disappeared when this compound(s) conjugated with Au NPs and showed enhanced activities. Furthermore, among the three popular antidiabetic standard drugs currently in the market, only acarbose inhibits both alpha glucosidase and alpha amylase. Vigliobose showed little effect on amylase whereas miglitol does not have any effects on amylase [90] Therefore, the high activity displayed by F1 on alpha-glucosidase and not for amylase agrees with the above studies. Furthermore, a close examination of the IC_50_ of LSTE, F1 and F2 for alpha-glucosidase indicates a similar functionality as mentioned above. Almost the same values were obtained for F1 and F2, which further supports the occurrence of the dimers and trimers of procyanidins, as rightly suggested by the NMR and LC–MS analysis. Procyanidins possessed similar arrangements of the hydroxyl groups as well as the aromatic ring systems and therefore, might have similar behaviors. However, the mechanism of action of alpha-glucosidase and alpha-amylase might not necessarily be the same. This has been demonstrated by the reports of many assays where different values were recorded for the two enzymes, even though the aim of inhibition is the same [91].

Au NPs of LSTE, F1 and F2 demonstrated activities that compete well with the intact extract/fractions. Although the activity of F1 Au NPs was identical to that of F1 for alpha-glucosidase, an enhanced alpha-amylase inhibition was recorded for F1 Au NPs. This behavior is similar to that reported by Shamprasad et al. [58], where Escin-Au NPs showed better activity compared to Escin in their study, thereby confirming our submission on the activity of F1 and F2 Au NPs. The activities in each case were almost two-fold of the corresponding precursor. This continuous resemblance of activity may stem from the starting materials of the nanoparticles. As earlier stated, F1 and F2 fractions are composed of dimer and trimer procyanidins as the major constituents. Therefore, closely related behaviors in the same experimental conditions are obvious. Accordingly, the improved antidiabetic performance of bimetallic Ag-Au NPs over their precursor extracts and acarbose has been reported [45,46]. The IC_50_ values of alpha-amylase on LSTE and its gold form also showed improved activity in agreement with previous studies [45]. The inhibiting powers of the nanomaterials may be a function of size and shape. As noted earlier, our Au NPs had a size range of 6–24 nm. These values are similar to the sizes in previous investigations [58]. Similarly, Niikura et al. [92] affirmed that spherical Au NPs of sizes 20 and 40 nm in diameter induced the west Nile virus better than those of other sizes and shapes. This may be the reason for an improved enzymatic activity of F1 and F2 Au NPs.

### 3.9. Antioxidant Activity/Total Phenolic Content

Oxidative stress has been linked to the cause of many deadly diseases like diabetes, the management of which is costly. Thus, plant extracts and nanoparticles with both antidiabetic and antioxidant properties will be greatly beneficial [93]. Therefore, the need to search for antioxidants with enhanced reducing abilities is crucial. Antioxidants are substances that can inhibit or delay the oxidation of a substrate when present in low concentrations. Due to the relationship of oxidative stress to other diseases, we, therefore, investigated the antioxidant capacities of LSTE, F1, F2 and the corresponding Au NPs. Three essays; Ferric reducing antioxidant power (FRAP), Folin–Ciocalteu (FC) and 2,2′-azino-bis-3-ethylbenzotiazolin-6- sulfonic acid (ABTS) were carried out and the results are presented in Table 3.

The mechanism with which FRAP operates is known as single electron transfer (SET), whereby an antioxidant transfers an electron to the corresponding cation, which would neutralize it [94]. In Table 3, strong activities were exhibited on FRAP with F1 displaying the highest activity (1834.0 ± 4.7 μM AAE/g). From the NMR and LC–MS analysis, F1 was found to contain a dimer and trimer of procyanidins that might have acted together in synergy to bring about this high activity. They possess hydroxyl groups attached to aromatic rings, which will perfectly participate in oxidation during the process. Similar activities were recorded for LSTE and F2 because they contained phenolic compounds of similar functionality. It is well known that phenolics have strong antioxidant activities [95]. The huge presence of many phenolics in LSTE has been demonstrated previously by LC–MS analysis, therefore its high activity is reasonable, while similar compounds, although different proportions were found in F2, accounted for its activity as well. However, since the FRAP’s mechanism is by electron transfer, the hydroxyl groups of the compound might be interacting with the nanoparticles, thereby limiting the site for the oxidation process. This may contribute to slightly reduced activity. In contrast, high activities in close ranges were demonstrated for all samples in FC assay except for LSTE Au NPs. Size, shape and the surrounding environment/medium is critical to the activity of nanoparticles. Owing to the type and number of phytochemicals taking part in the fabrication of LSTE Au NPs, there is a large size difference from those of F1 and F2 and the stability was also slightly higher. Therefore, it is expected to behave somewhat differently since the smaller sized particles have a higher surface area. In addition, more phytochemicals are present in LSTE, which creates a different surrounding environment for the NPs. Previously, nanoparticles with smaller sizes were reported to show enhanced activity in comparison to relatively larger ones [96]. On the other hand, a clear trend can be observed for the ABTS assay. This is probably because of the difference in the mechanism of operation between the assays. ABTS is largely operating on hydrogen atom transfer (HAT). The trend in the ABTS results is such that individual Au NPs demonstrated better antioxidant capacity relative to their respective precursors. Recent research reports [54,57,58,95] supported the above submission. Au NPs biosynthesized from *Halymenia dilatata* also demonstrated higher antioxidant activity than the starting plant extract [97].

However, results of antioxidant activities often vary from one assay to the other, probably because of the difference in mechanism of operation where some operate by SET or HAT or both. The nature of the sample, the medium of operation and the functionality might account for the variations in our results from one assay to the other (Table 3).

### 3.10. Quantification of the Total Phenolic Content in the Au NPs from the FC Assay

Results from Table 3 revealed the phenolic content of the intact fractions (F1 and F2) and the total extracts, as well as the phenolic content (FC) of the corresponding Au NPs. According to the HRTEM and DLS analysis, the average diameter was smaller in case of the LSTE Au NPs relative to the others. Hence, the higher surface area available for interaction with the compounds may explain the smaller phenolic content (29.3%) observed. However, an increase in the percentage FC was observed in the case of F1 and F2, with maximum concentration of F2 (76.3%). The high payload of F2 may be explained in terms of the homogeneity of the compound in that fraction as well as its high packing power.

## 4. Conclusions

The preliminary screening of the *L. sericea* total extract showed the potential of generating stable Au NPs. An intent made to purify the pure compound(s) responsible for such activity resulted in the purification of two inseparable procyanidins fractions namely F1 and F2. The first fraction, F1, contained a mixture of procyanidins dimer (B series) and trimers, while the second fraction, F2, contained four major procyanidin dimers belonging to B series. These fractions successfully formed stable Au NPs confirming the ability of these class of phytochemicals to act as reducing agents. DLS measurements and in vitro stability examinations further affirmed their stability in physiological conditions without the introduction of any external stabilizers. This means that the compounds also doubled as capping agents that prevented agglomeration for the given period. Thereafter, biological activities were carried out. LSTE constituents and hybrid nanoparticles showed interesting inhibitory activities on both alpha-glucosidase and alpha-amylase at low concentrations. F1 and its Au NPs demonstrated enhanced alpha-glucosidase activities compared to LSTE and LSTE Au NPs. For alpha-amylase, F2 Au NPs showed the highest inhibitory activities, which were another interesting behavior that calls for further attention. The particles also exhibited interesting antioxidant activity thereby buttressing their potential biological applications since this activity has been linked to some diseases. As far as we know, this work is the first scientific report on the identification of procyanidins from the aerial parts of *Leucosidea sericea*, and the use thereof in nanoparticle synthesis. The results suggest that procyanidins had the ability to reduce gold to form biostable and bioactive gold nanoparticles with potential antidiabetic and antioxidant applications.

## Figures and Tables

**Table 1:** Particle size and zeta potential for Au NPs obtained from DLS.

Sample	Hydrodynamic Size (nm)	Zeta Potential (mV)
LSTE Au NPs	21.85	−33.9
F1 Au NPs	27.20	−32.5
F2 Au NPs	26.91	−29.1

**Table 2:** Inhibitory activities of LSTE, F1, F2 and Au NPs on alpha-glucosidase and alpha-amylase.

Items	Alpha-Glucosidase IC_50_ (µg/mL)	Alpha-Amylase IC_50_ (µg/mL)
LSTE	8.1 ± 0.6	3.5 ± 0.7
LSTE Au NPs	14.5 ± 0.8	3.0 ± 0.3
F1	7.3 ± 0.5	NA
F1 Au NPs	7.3 ± 0.3	1.8 ± 0.3
F2	7.1 ± 0.4	18.9 ± 0.2
F2 Au NPs	4.5 ± 0.6	10.5 ± 0.1
Acarbose	610 ± 2.6	10.2 ± 0.6

NA: not active at the tested concentrations, Acarbose: positive control.

**Table 3:** Antioxidant activities of F 2, LSTE, F 1 and the corresponding Au NPs.

Items	FRAP (μM AAE/g)	ABTS (μM TE/g)	FC (μM GAE/g)	FC% (Au NPs)
LSTE	1113.2 ± 6.7	814.9 ± 6.1	602.6 ± 6.1	
LSTE Au NPs	113.8 ± 9.5	1059.4 ± 7.4	179.8 ± 6.2	29.3
F1	1834.0 ± 4.7	818.2 ± 7.7	889.6 ± 6	
F1 Au NPs	748.6 ± 1.4	1521.9 ± 3.0	356.7 ± 6.6	40.1
F2	1166.0 ± 2.1	816.9 ± 8.6	685.7 ± 6.7	
F2 Au NPs	1083.8 ±1.2	861.9 ± 5.3	523.1 ± 4.4	76.3
Standard	3976.8 ± 3.8 *	7525.0 ± 4.9 **		

* = vitamin C, ** = trolox. The results are expressed as mean ± SD for *n* = 3.

## Supplementary Materials

The following are available online at https://www.mdpi.com/2218-273X/10/3/452/s1, Figure S1: 1H NMR of Fraction F1; S2: 13C NMR of Fraction F1; S3: 13C –DEPT-135 NMR of Fraction F1; S4: 1H NMR of Fraction F2; S5: 13C NMR of Fraction F2; S6: 13C –DEPT-135 NMR of Fraction F2.

## References

[1] Duan H., Wang D., Li Y. (2015). Green chemistry for nanoparticle synthesis. Chem. Soc. Rev..

[2] Saravanakumar A., Peng M.M., Ganesh M., Jayaprakash J., Mohankumar M., Jang H.T. (2017). Low-cost and eco-friendly green synthesis of silver nanoparticles using *Prunus japonica* (Rosaceae) leaf extract and their antibacterial, antioxidant properties. Artif. Cells Nanomed. Biotechnol..

[3] Jha A.K., Prasad K. (2010). Green synthesis of silver nanoparticles using Cycas leaf. Int. J. Green Nanotechnol. Phys. Chem..

[4] Liu J., Qin G., Raveendran P., Ikushima Y. (2006). Facile “green” synthesis, characterization, and catalytic function of β-D-glucose-stabilized Au nanocrystals. Chem. Eur. J..

[5] Kumar V., Mohan S., Singh D.K., Verma D.K., Singh V.K., Hasan S.H. (2017). Photo-mediated optimized synthesis of silver nanoparticles for the selective detection of Iron (III), antibacterial and antioxidant activity. Mater. Sci. Eng. C.

[6] Tekale S.U., Kauthale S.S., Pagore V.P., Jadhav V.B., Pawar R.P. (2013). ZnO nanoparticle-catalyzed efficient one-pot three-component synthesis of 3, 4, 5-trisubstituted furan-2 (5*H*)-ones. J. Iran. Chem. Soc..

[7] Ergin A.D., Bayindir Z.S., Yüksel N. (2019). Characterization and optimization of colon targeted S-adenosyl-L-methionine loaded chitosan nanoparticles. Marmara Pharm. J..

[8] Liu Y., Welch M.J. (2012). Nanoparticles labeled with positron emitting nuclides: Advantages, methods, and applications. Bioconj. Chem..

[9] Xie Y., Kocaefe D., Chen C., Kocaefe Y. (2016). Review of research on template methods in preparation of nanomaterials. J. Nanomater..

[10] Singh P., Kim Y.J., Zhang D., Yang D.C. (2016). Biological synthesis of nanoparticles from plants and microorganisms. Trends Biotechnol..

[11] Lysy P.A., Corritore E., Sokal E.M. (2016). New insights into diabetes cell therapy. Curr. Diabetes Rep..

[12] Renner S., Blutke A., Clauss S., Deeg C.A., Kemter E., Merkus D., Wanke R., Wolf E. (2020). Porcine models for studying complications and organ crosstalk in diabetes mellitus. Cell Tissue Res..

[13] Cho N., Shaw J.E., Karuranga S., Huang Y., da Rocha Fernandes J.D., Ohlrogge A.W., Malanda B. (2018). IDF diabetes atlas: Global estimates of diabetes prevalence for 2017 and projections for 2045. Diabetes Res. Clin. Pract..

[14] Lorenzati B., Zucco C., Miglietta S., Lamberti F., Bruno G. (2010). Oral hypoglycemic drugs: Pathophysiological basis of their mechanism of action. Pharmaceuticals.

[15] Santos M.S.C., Azevedo R.B., Teixeira P.R., Sales M.J.A., Báo S.N., Paterno L.G., Silva A.L.G. (2016). Photochemically-assisted synthesis of non-toxic and biocompatible gold nanoparticles. Colloids Surf. B Biointerfaces.

[16] Ahmad T., Azmi M., Irfan M., Moniruzzaman M., Asghar A., Bhattacharjee S. (2018). Green synthesis of stabilized spherical shaped gold nanoparticles using novel aqueous Elaeis guineensis (Oil palm) leaves extract. J. Mol. Struct..

[17] Zhang G., Li Y., Gao X. (2018). An asynchronous-alternating merging-zone flow-injection gold nanoparticles probe method for determination of Antidiabetic pioglitazone hydrochloride medicine. New J. Chem..

[18] Yilmazer-Musa M., Griffith A.M., Michels A.J., Schneider E., Frei B. (2012). Grape seed and tea extracts and catechin 3-gallates are potent inhibitors of α-amylase and α-glucosidase activity. J. Agric. Food Chem..

[19] Tanko Y., Yerima M., Mahdi M.A., Yaro A.H., Musa K.Y., Mohammed A. (2008). Hypoglycemic activity of methanolic stem bark of adansonnia digitata extract on blood glucose levels of streptozocin-induced diabetic wistar rats. Int. J. Appl. Res. Nat. Prod..

[20] Van de Venter M., Roux S., Bungu L.C., Louw J., Crouch N.R., Grace O.M., Folb P. (2008). Anti-diabetic screening and scoring of 11 plants traditionally used in South Africa. J. Ethnopharmacol..

[21] Eidi A., Eidi M., Esmaeili E. (2006). Anti-diabetic effect of garlic (Allium sativum L.) in normal and streptozotocin-induced diabetic rats. Phytomedicine.

[22] Rizvi M.M.A., El Hassadi I.M.G., Younis S.B. (2009). Bioefficacies of Cassia fistula: An Indian labrum. Afr. J. Pharm. Pharmacol..

[23] Sethi J., Sood S., Seth S., Talwar A. (2004). Evaluation of hypoglycemic and antioxidant effect of *Ocimum sanctum*. Indian J. Clin. Biochem..

[24] Nammi S., Boini M., Lodagala S., Behara R. (2003). The juice of fresh leaves of *Catharanthus roseus* Linn. reduces blood glucose in normal and alloxan diabetic rabbits. BMC Complement. Altern. Med..

[25] Yagi A., Hegazy S., Kabbash A., Abd-El Wahab E. (2009). Possible hypoglycemic effect of *Aloe vera* L. high molecular weight fractions on type 2 diabetic patients. Saudi Pharm. J..

[26] Yalçın S., Erkan M., Ünsoy G., Parsian M., Kleeff J., Gündüz U. (2014). Effect of gemcitabine and retinoic acid loaded PAMAM dendrimer-coated magnetic nanoparticles on pancreatic cancer and stellate cell lines. BioMed Pharm..

[27] Kaleem M., Sheema S.H., Bano B. (2005). Protective effects of *Piper nigrum* and *Vinca rosea* in alloxan induced diabetic rats. Indian J. Physiol. Pharmacol..

[28] Ponnanikajamideen M., Rajeshkumar S. (2019). In vivo type 2 diabetes and wound-healing effects of antioxidant gold nanoparticles synthesized using the insulin plant *Chamaecostus cuspidatus* in albino rats. Can. J. Diabetes.

[29] Khalil M. (2016). Biosynthesis of gold nanoparticles using extract of grape (*Vitis vinifera*) leaves and seeds. Prog. Nanotechnol. Nanomater..

[30] Daisy P., Saipriya K. (2012). Biochemical analysis of *Cassia fistula* aqueous extract and phytochemically synthesized gold nanoparticles as hypoglycemic treatment for diabetes mellitus. Int. J. Nanomed..

[31] John P.A., Zhijian C., Naidu M. (2015). Neurite outgrowth stimulatory effects of myco¬synthesized Au NPs from *Hericium erinaceus* (Bull.: Fr.) Pers. on pheochromocytoma (PC-12) cells. Int. J. Nanomed..

[32] Venkatraman A., Yahoob S.A.M., Nagarajan Y., Harikrishnan S., Vasudevan S., Murugasamy T. (2018). Pharmacological activity of biosynthesized gold nanoparticles from brown algae-*Seaweed turbinaria* conoides. Nanowrold J..

[33] Opris R., Tatomir C., Olteanu D., Moldovan R., Moldovan B., David L., Adriana G. (2017). The effect of Sambucus nigra L. extract and phytosinthesized gold nanoparticles on diabetic rats. Colloids Surf. B Biointerfaces.

[34] Dhas T.S., Kumar V.G., Karthick V., Vasanth K., Singaravelu G., Govindaraju K. (2016). Enzyme and microbial technology effect of biosynthesized gold nanoparticles by *Sargassum swartzii* in alloxan induced diabetic rats. Enzyme Microb. Technol..

[35] Malapermal V., Mbatha N., Gengan R., Anand K. (2015). Biosynthesis of bimetallic Au-Ag nanoparticles using *Ocimum basilicum* (L.) with anti-diabetic and antimicrobial properties. Adv. Mater. Lett..

[36] Elobeid M.A. (2016). Amelioration of streptozotocin induced diabetes in rats by eco-friendly composite nano-cinnamon extract. Pak. J. Zool..

[37] Ovais M., Khalil A.T., Islam N.U., Ahmad I., Ayaz M., Saravanan M., Mukherjee S. (2018). Role of plant phytochemicaLSTE and microbial enzymes in biosynthesis of metallic nanoparticles. Appl. Microbiol. Biotechnol..

[38] Khan M.A., Raza A., Ovais M., Sohail M.F., Ali S. (2018). Current state and prospects of nano-delivery systems for sorafenib. Int. J. Polym. Mater. Polym. Biomater..

[39] Ali M., Khan T., Fatima K., Aliqul A., Ovais M., Khalil A.T., Idrees M. (2018). Selected hepatoprotective herbal medicines: Evidence from ethnomedicinal applications, animal models and possible mechanism of actions. Phytother. Res..

[40] Aromal S.A., Vidhu V.K., Philip D. (2012). Green synthesis of well-dispersed gold nanoparticles using *Macrotyloma uniflorum*. Spectrochim. Acta Part A.

[41] Edison T.J.I., Sethuraman M.G. (2012). Instant green synthesis of silver nanoparticles using *Terminalia chebula* fruit extract and evaluation of their catalytic activity on reduction of methylene blue. Process Biochem..

[42] Mohan Kumar K., Mandal B.K., Sinha M., Krishnakumar V. (2012). *Terminalia chebula* mediated green and rapid synthesis of gold nanoparticles. Spectrochim. Acta Part A.

[43] Stephen A., Seethalakshmi S. (2013). Phytochemical synthesis and preliminary characterization of silver nanoparticles using hesperidin. J. Nanosci..

[44] Sahu N., Soni D., Chandrashekhar B., Satpute D.B., Saravanadevi S., Sarangi B.K., Pandey R.A. (2016). Synthesis of silver nanoparticles using flavonoids: Hesperidin, naringin and diosmin, and their antibacterial effects and cytotoxicity. Int. Nano Lett..

[45] Bisht S., Feldmann G., Soni S., Ravi R., Karikar C., Maitra A., Maitra A. (2007). Polymeric nanoparticle-encapsulated curcumin (‘nanocurcumin’): A novel strategy for human cancer therapy. J. Nanobiotechnol..

[46] Khaleel S., Govindaraju K., Manikandan R., Seog J., Young E., Singaravelu G. (2010). Phytochemical mediated gold nanoparticles and their PTP 1B inhibitory activity. Colloids Surf. B Biointerfaces.

[47] Payne J.N., Badwaik V.D., Waghwani H.K., Moolani H.V., Tockstein S., Thompson D.H., Dakshinamurthy R. (2018). Development of dihydrochalcone-functionalized gold nanoparticles for augmented antineoplastic activity. Int. J. Nanomed..

[48] Shamprasad B.R., Keerthana S., Megarajan S., Lotha R., Aravind S., Veerappan A. (2019). Photosynthesized escin stabilized gold nanoparticles exhibit anti-diabetic activity in L6 rat skeletal muscle cells. Mater. Lett..

[49] Dong Y., Wan G., Yan P., Qian C., Li F., Peng G. (2019). Biology fabrication of resveratrol coated gold nanoparticles and investigation of their effect on diabetic retinopathy in streptozotocin induced diabetic rats. J. Photochem. Photobiol. B Biol..

[50] Rajarajeshwari T., Shivashri C., Rajasekar P. (2014). Synthesis and characterization of biocompatible gymnemic acid—Gold nanoparticles: A study on glucose uptake stimulatory effect in 3T3-L1 adipocytes. RSC Adv..

[51] Bhumkar D.R., Joshi H.M., Sastry M., Pokharkar V.B. (2007). Chitosan reduced gold nanoparticles as novel carriers for transmucosal delivery of insulin. Pharm. Res..

[52] Cho H.J., Oh J., Choo M.K., Ha J.I., Park Y., Maeng H.J. (2014). Chondroitin sulfate-capped gold nanoparticles for the oral delivery of insulin. Int. J. Biol. Macromol..

[53] Dubey K., Anand B.G., Badhwar R., Bagler G. (2015). Tyrosine and tryptophan coated gold nanoparticles inhibit amyloid aggregation of insulin. Amino Acids.

[54] Khoshnamvand M., Ashtiani S., Huo C., Saeb S.P., Liu J. (2019). Use of Alcea rosea leaf extract for biomimetic synthesis of gold nanoparticles with innate free radical scavenging and catalytic activities. J. Mol. Struct..

[55] Pu S., Li J., Sun L., Zhong L., Ma Q. (2019). An in vitro comparison of the antioxidant activities of chitosan and green synthesized gold nanoparticles. Carbohydr. Polym..

[56] Torabi N., Nowrouzi A., Ahadi A., Vardasbi S., Etesami B. (2019). Green synthesis of gold nanoclusters using seed aqueous extract of Cichorium intybus L. and their characterization. SN Appl. Sci..

[57] Nakkala J.R., Bhagat E., Suchiang K., Sadras S.R. (2015). Comparative study of antioxidant and catalytic activity of silver and gold nanoparticles synthesized from Costus pictus leaf extract. J. Mater. Sci. Technol..

[58] Benedec D., Oniga I., Cuibus F., Sevastre B., Stiufiuc G., Duma M., Lucaciu C.M. (2018). Origanum vulgare mediated green synthesis of biocompatible gold nanoparticles simultaneously possessing plasmonic, antioxidant and antimicrobial properties. Int. J. Nanomed..

[59] Zhaleh M., Zangeneh A., Goorani S., Seydi N., Zangeneh M.M., Tahvilian R., Pirabbasi E. (2019). In vitro and in vivo evaluation of cytotoxicity, antioxidant, antibacterial, antifungal, and cutaneous wound healing properties of gold nanoparticles produced via a green chemistry synthesis using Gundelia tournefortii L. as a capping and reducing agent. Appl. Organomet. Chem..

[60] Patil M.P., Seo Y.B., Lim H.K., Kim G.D. (2019). Biofabrication of gold nanoparticles using Agrimonia pilosa extract and their antioxidant and cytotoxic activity. Green Chem. Lett. Rev..

[61] Veena S., Devasena T., Sathak S.S.M., Yasasve M., Vishal L.A. (2019). Green Synthesis of gold nanoparticles from vitex negundo leaf extract: Characterization and in vitro evaluation of antioxidant-antibacterial activity. J. Clust. Sci..

[62] Bharathi D., Bhuvaneshwari V. (2019). Evaluation of the cytotoxic and antioxidant activity of phyto-synthesized silver nanoparticles using Cassia angustifolia flowers. J. Bionanosci..

[63] Zayed M.F., Mahfoze R.A., El-kousy S.M., Al-Ashkar E.A. (2020). In-vitro antioxidant and antimicrobial activities of metal nanoparticles biosynthesized using optimized Pimpinella anisum extract. Colloid Surf. A Phys. Eng..

[64] Aremu A.O., Amoo S.O., Ndhlala A.R., Finnie J.F., Van Staden J. (2011). Antioxidant activity, acetylcholinesterase inhibition, iridoid content and mutagenic evaluation of *Leucosidea sericea*. Food Chem. Toxicol..

[65] Pendota S.C., Aremu A.O., Slavětínská L.P., Rárová L., Grúz J., Doležal K., Van Staden J. (2018). Identification and characterization of potential bioactive compounds from the leaves of *Leucosidea sericea*. J. Ethnopharmacol..

[66] Nair J.J., Aremu A.O., Van Staden J. (2012). Anti-inflammatory effects of *Leucosidea sericea* (Rosaceae) and identification of the active constituents. S. Afr. J. Bot..

[67] Mafole T.C., Aremu A.O., Mthethwa T., Moyo M. (2017). An overview on *Leucosidea sericea* Eckl. & Zeyh.: A multi-purpose tree with potential as a phytomedicine. J. Ethnopharmacol..

[68] Adamu M., Mukandiwa L., Awouafack M.D., Ahmed A.S., Eloff J.N., Naidoo V. (2019). Ultrastructure changes induced by the phloroglucinol derivative agrimol G isolated from *Leucosidea sericea* in Haemonchus contortus. Exp. Parasitol..

[69] Sharma R., Kishore N., Hussein A., Lall N. (2014). The potential of *Leucosidea sericea* against *Propionibacterium acnes*. Phytochem. Lett..

[70] Bosman A.A., Combrinck S., Roux-Van der Merwe R., Botha B.M., McCrindle R.I., Houghton P.J. (2004). Isolation of an anthelmintic compound from *Leucosidea sericea*. S. Afr. J. Bot..

[71] Stander M.A., Van Wyk B.E., Taylor M.J., Long H.S. (2017). Analysis of phenolic compounds in rooibos tea (Aspalathus linearis) with a comparison of flavonoid-based compounds in natural populations of plants from different regions. J. Agric. Food Chem..

[72] Elbagory A.M., Meyer M., Cupido C.N., Hussein A.A. (2017). Inhibition of bacteria associated with wound infection by biocompatible green synthesized gold nanoparticles from South African plant extracts. Nanomaterials.

[73] Ademiluyi A.O., Oboh G. (2013). Experimental and toxicologic pathology soybean phenolic-rich extracts inhibit key-enzymes linked to type 2 diabetes (α-amylase and α-glucosidase) and hypertension (angiotensin I converting enzyme) in vitro. Exp. Toxicol. Pathol..

[74] Jeremia L. (2014). Inhibitory effects of five medicinal plants on rat alpha-glucosidase: Comparison with their effects on yeast alpha-glucosidase. J. Med. Plant Res..

[75] Ranjan Sarker S., Polash S.A., Boath J., Kandjani A.E., Poddar A., Dekiwadia C., Bhargava S.K. (2019). Functionalization of elongated tetrahexahedral Au nanoparticles and their antimicrobial activity assay. ACS Appl. Mater. Interfaces.

[76] Salar R.K., Certik M., Brezova V. (2012). Modulation of phenolic content and antioxidant activity of maize by solid state fermentation with Thamnidium elegans CCF 1456. Biotechnol. Bioprocess Eng..

[77] Arts M.J., Haenen G.R., Voss H.P., Bast A. (2004). Antioxidant capacity of reaction products limits the applicability of the Trolox Equivalent Antioxidant Capacity (TEAC) assay. Food Chem. Toxicol..

[78] Re R., Pellegrini N., Proteggente A., Pannala A., Yang M., Rice-Evans C. (1999). Antioxidant activity applying an improved ABTS radical cation decolorization assay. Free Radic. Biol. Med..

[79] Raveendran P., Fu J., Wallen S.L. (2003). Completely ‘Green’ synthesis and stabilization of metal nanoparticles. J. Am. Chem. Soc..

[80] Elbagory A.M., Cupido C.N., Meyer M., Hussein A.A. (2016). Large scale screening of southern African plant extracts for the green synthesis of gold nanoparticles using microtitre-plate method. Molecules.

[81] Elia P., Zach R., Hazan S., Kolusheva S., Porat Z., Zeiri Y. (2014). Green synthesis of gold nanoparticles using plant extracts as reducing agents. Int. J. Nanomed..

[82] Chen R., Wu J., Li H., Cheng G., Lu Z., Che C.M. (2010). Fabrication of gold nanoparticles with different morphologies in HEPES buffer. Rare Metals.

[83] Foss C.A., Hornyak G.L., Stockert J.A., Martin C.R. (1994). Template-synthesized nanoscopic gold particles: Optical spectra and the effects of particle size and shape. J. Phys. Chem..

[84] Fang C., Ma Z., Chen L., Li H., Jiang C., Zhang W. (2019). Biosynthesis of gold nanoparticles, characterization and their loading with zonisamide as a novel drug delivery system for the treatment of acute spinal cord injury. J. Photochem. Photobiol. B Biol..

[85] Krishnamurthy S., Esterle A., Sharma N.C., Sahi S.V. (2014). Yucca-derived synthesis of gold nanomaterial and their catalytic potential. Nanoscale Res. Lett..

[86] Brewer S.H., Glomm W.R., Johnson M.C., Knag M.K., Franzen S. (2005). Probing BSA binding to citrate-coated gold nanoparticles and surfaces. Langmuir.

[87] Thilagam E., Parimaladevi B., Kumarappan C., Mandal S.C. (2013). a-Glucosidase and a-Amylase inhibitory activity of senna surattensis. J. Acupunct. Meridian Stud..

[88] Sofowora A., Ogunbodede E., Onayade A., Dentistry C. (2013). The role and place of medicinal plants in the strategies for disease. J. Afr. Tradit. Complement..

[89] Etsassala N.G., Badmus J.A., Waryo T.T., Marnewick J.L., Cupido C.N., Hussein A.A., Iwuoha E.I. (2019). Alpha-glucosidase and alpha-amylase inhibitory activities of novel abietane diterpenes from *Salvia africana-lutea*. Antioxidants.

[90] Coman C., Rugină O.D., Socaciu C. (2012). Plants and natural compounds with antidiabetic action. Not. Bot. Horti Agrobo.

[91] Niikura K., Matsunaga T., Suzuki T., Kobayashi S., Yamaguchi H., Orba Y., Sawa H. (2013). Gold nanoparticles as a vaccine platform: Influence of size and shape on immunological responses in vitro and in vivo. ACS Nano.

[92] Virk P. (2018). Anti-diabetic activity of green gold-silver nanocomposite with trigonella foenum graecum l. seeds extract on streptozotocin-induced diabetic rats. Pak. J. Zool..

[93] Perez-Fons L., GarzÓn M.T., Micol V. (2009). Relationship between the antioxidant capacity and effect of rosemary (Rosmarinus officinalis L.) polyphenoLSTE on membrane phospholipid order. J. Agric. Food Chem..

[94] Duletić-Laušević S., Aradski A.A., Kolarević S., Vuković-Gačić B., Oalđe M., Živković J., Šavikin K., Marin P.D. (2018). Antineurodegenerative, antioxidant and antibacterial activities and phenolic components of *Origanum majorana* L.(Lamiaceae) extracts. J. Appl. Bot. Food Qual..

[95] BarathManiKanth S., Kalishwaralal K., Sriram M., Pandian S.R.K., Youn H.S., Eom S., Gurunathan S. (2010). Anti-oxidant effect of gold nanoparticles restrains hyperglycemic conditions in diabetic mice. J. Nanobiotechnol..

[96] Ajitha B., Reddy Y.A.K., Reddy P.S. (2015). Enhanced antimicrobial activity of silver nanoparticles with controlled particle size by pH variation. Powder Technol..

[97] Vinosha M., Palanisamy S., Muthukrishnan R., Selvam S., Kannapiran E., You S., Prabhu N.M. (2019). Biogenic synthesis of gold nanoparticles from Halymenia dilatata for pharmaceutical applications: Antioxidant, anti-cancer and antibacterial activities. Process Biochem..

